# Supplementary material for: Detecting structural heterogeneity in single-molecule localization microscopy data
Source: Nat Commun. 2021 Jun 18;12:3791. doi: 10.1038/s41467-021-24106-8 (PMC8213809; doi:10.1038/s41467-021-24106-8)
Supplement: Supplementary file 1 — Supplementary Information [file 41467_2021_24106_MOESM1_ESM.pdf]

Supplementary Information

Detecting structural heterogeneity in single-molecule localization microscopy data

Teun A.P.M. Huijben, Hamidreza Heydarian, Alexander Auer, Florian Schueder, Ralf Jungmann, Sjoerd Stallinga and Bernd Rieger

Contents

Supplementary Note 1: **Hierarchical agglomerative clustering as alternative classification approach** **3**

Supplementary Figure 1: **Determine value of *K* from scatter plot containing the first three dimensions of the MDS space** **5**

Supplementary Figure 2: **Determine value of *K* using the silhouette cluster evaluation measure** **6**

Supplementary Figure 3: **Distributions of number of localizations per particle for digits datasets** **7**

Supplementary Figure 4: **Reconstruction of pure classes of digits dataset** **8**

Supplementary Figure 5: **Fourier ring correlation measurements for the digits reconstructions** **9**

Supplementary Figure 6: **Justification for using four classes to capture the misfolds** **10**

Supplementary Figure 7: **Classification into 5 classes needed to capture the letter L** **11**

Supplementary Figure 8: **Detecting rare classes of 9-fold simulated nuclear pores** **12**

Supplementary Figure 9: **Ellipticity of NPC integral membrane protein gp210** **13**

Supplementary Figure 10: **Classification of multiple simulated dataset** **14**

Supplementary Figure 11: **Classification on 3D DNA-origami tetrahedron structure reveals variation in height due to folding variability** **15**

Supplementary Figure 12: **Comparison between the MDS and HAC approach** **16**

Supplementary Figure 13: **Optimal number of dimensions in multidimensional scaling** **17**

Supplementary Figure 14: **Optional further classification for datasets with small subgroups of structurally different particles** **18**

Supplementary Tab. 1: **M13mp18 p7249 sequence** 19
Supplementary Tab. 2: **Rectangular DNA origami staple strands** 20
Supplementary Tab. 3: **Biotinylated staple strands** 24
Supplementary Tab. 4: **DNA-PAINT docking site sequences** 24
Supplementary Tab. 5: **DNA-PAINT imager sequences** 24
Supplementary Tab. 6**: Experimental conditions for sample Letter O** 25
Supplementary Tab. 7: **Experimental conditions for sample Letter T** 25
Supplementary Tab. 8: **Experimental conditions for sample Letter L** 25
Supplementary Tab. 9: **Experimental conditions for combined sample Letter O, Letter T, Letter L** 25

Supplementary Note 1: Hierarchical agglomerative clustering as alternative classification approach

The classification pipeline (**Fig. 1**) is based on the dissimilarities between all the particles. In general, there are two techniques available to cluster based on pairwise dissimilarities: clustering based on a spatial embedding of the particles and direct hierarchical clustering. We have implemented and optimized both strategies and tested them on multiple datasets. The performance of the first method, i.e. clustering based on a spatial embedding, performs significantly better than hierarchical clustering. Therefore, the first method, hereafter referred to as the multidimensional scaling (MDS) approach, is used for all classifications in this paper.

In this supplementary note, we will explain the implementation and results of hierarchical clustering. Although it has not been tested extensively on all available datasets, we will show its capabilities and compare the performance with the established multidimensional scaling approach.

The HAC algorithm

Hierarchical clustering is a method that can cluster objects based on a built hierarchy. The hierarchy is visualized in a dendrogram (**Supplementary Fig. 15a**), which is a tree-like graph that indicates the distances between all clusters. Dendrograms are commonly used in phylogeny, to study the evolutionary history among organisms.

Types of clustering

There are two types of hierarchical clustering: agglomerative and divisive. In hierarchical agglomerative clustering, every object is initially seen as an individual cluster. The dendrogram is constructed by iteratively merging the two closest clusters until all objects are part of the same cluster. In hierarchical divisive clustering, all objects start as part of one cluster, which is iteratively split until all clusters contain one object. We use the agglomerative approach since we are dealing with thousands of particles, and for large N , the computational complexity for agglomerative clustering is lower than for divisive clustering.

Distance criterion

It is not trivial to define the distance between two clusters since only the pairwise distances between the objects are available. There are multiple criteria that can be used as a metric for the distance between two clusters, the most common variants are the single-, average- and complete-linkage criteria. With the single-linkage criterion, the distance between two clusters is defined as the shortest distance between any two objects in these two clusters. In each step of building the dendrogram, the two clusters are merged that contain the closest pair of objects that are not yet belonging to the same cluster. This strategy tends to find elongated clusters, since adding a point to an existing cluster only depends on the shortest distance to that cluster, not on the distances to all the other points. The complete-linkage criterion defines the distance between two clusters as the longest distance between any two objects in these two clusters. This strategy tends to find spherical, compact clusters, since adding a point to an existing cluster depends on the distance to the furthest point within that cluster. A third criterion is average-linkage, where the distance between two clusters is defined as the average distance between all pairs of objects. This is the most robust criterion, since the distance between to clusters depends on all pairwise distances among objects.

Input for the hierarchical clustering

We start with an upper-triangular matrix of $N(N-1)/2$ dissimilarity values for N particles. The dissimilarity values result from the Bhattacharya cost function values obtained with all-to-all registration. The cost function gives similarity values, $S(a,b)$, which are converted to dissimilarity values, $D(a,b)$, by subtracting them from the highest value in the matrix: $D(a,b) = \max(S)\text{-}S(a,b)$. The dissimilarity values are used as the distances between the particles in the hierarchical agglomerative clustering and are the same values that are used as input for the multidimensional scaling in the main approach.

Clustering

The constructed dendrogram is used for clustering of the particles. Since the dendrogram represents the hierarchy for the particles, the clustering is performed by defining a threshold and pruning of the dendrogram (**Supplementary Fig. 15a**). The particles of each pruned sub-dendrogram belong to one cluster and can subsequently be reconstructed per cluster. The bottleneck of this clustering approach is determining the threshold for pruning the dendrogram.

Comparison of the HAC to the multidimensional scaling approach

The HAC approach for clustering the particles is tested on multiple datasets. It is clear that the MDS approach performs significantly better than HAC, especially for the more challenging datasets. Here, we will show two examples where MDS outperforms HAC.

Example 1: Manually flipped experimental TUD-logos (50% DoL)

For the first example, we used the experimental dataset of DNA-origami TUD-logos imaged with DNA-PAINT (**Fig. 3d**). The dataset contained 440 particles in two classes, of which 50% are intentionally flipped by image processing, so 220 particles have the normal orientation, and 220 particles are mirrored. The dissimilarity values were obtained with all-to-all registration and used by HAC and MDS to cluster the particles. The dendrograms for average- and complete-linkage (**Supplementary Fig. 15b-c**) created by the HAC approach show some grouping of the classes, but the classes are fragmented into many groups. This makes proper clustering impossible, since no threshold value exists to prune the dendrogram into the correct classes. On the contrary, the first two dimensions of the MDS approach (**Supplementary Fig. 15d**) show clear grouping of the two classes. Subsequent k-means clustering of the MDS space results in correct clustering for 95% of the particles, which shows that the MDS approach is able to better classify the images than the HAC method.

Example 3: Experimental four-class nanoTRON dataset

As a second example, we examined the classification performance of HAC and MDS on the digits dataset (**Fig. 2b-f**). The dataset used here contained 200 particles, 50 per class, which were imaged separately per class. The dendrograms created by HAC show clustering of the classes, however, the architecture of the dendrogram does not show a clear pruning threshold that will separate all four classes correctly (**Supplementary Fig. 15e-f**). Contrary to HAC, the first dimensions of the MDS approach show a clear clustering of the particles per class (**Supplementary Fig. 15g**). With subsequent k-means clustering, the particles can be classified with a 93% performance.

Conclusion

In conclusion, easy-to-classify data can be classified correctly by both HAC and MDS, although HAC does require the delicate choice of the right parameters, like the distance criterion and the pruning threshold. The two examples show that these datasets can only be classified correctly using the MDS approach and not by HAC. Other advantages of the MDS approach are that there is no choice for the linkage criterion and that no particles are lost in pruning the tree, because of the creation of single-particle clusters.

The MDS approach still requires choosing the number of K clusters in k-means clustering, however, we give a detailed description on how to determine the optimal value of K (see main text). The choice of K is significantly less delicate than the choice of the pruning parameter in the HAC approach. A possible reason for the better performance of the MDS approach could be that the multidimensional embedding of the particles in the MDS approach allows for more flexibility than the hierarchy captured in a dendrogram. In principle, all dissimilarity values from the all-to-all registration matrix are used and preserved in the multidimensional embedding, whereas the hierarchical clustering can be too dependent on certain particles, since one dissimilarity value can have a major effect on the inter-cluster distance. The multidimensional scaling effectively performs dimensionality reduction of the dissimilarity space, removing the noise that results from individual misregistrations.

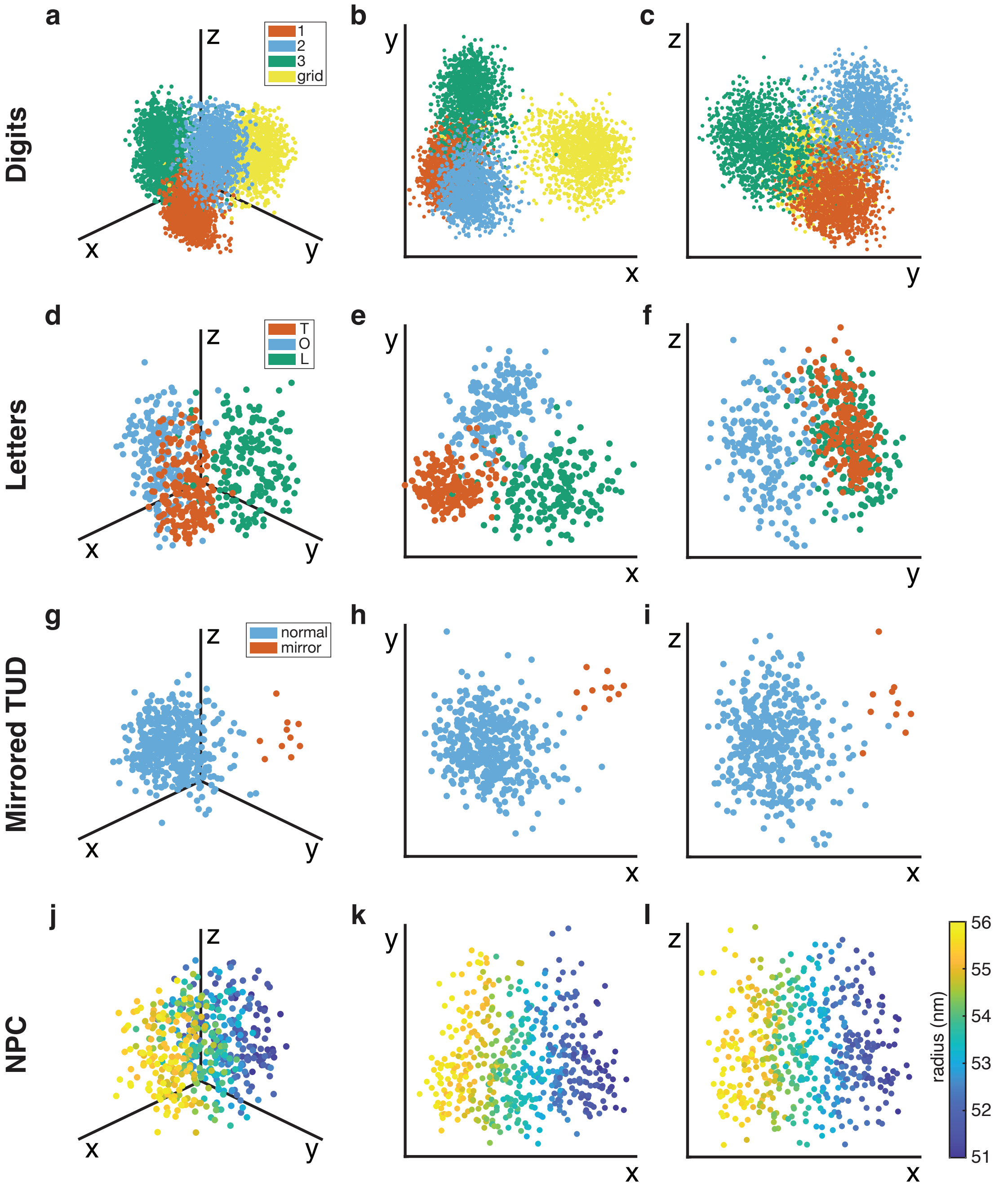

Supplementary Fig. 1 | Determine value of *K* from scatter plot containing the first three dimensions of the MDS space. Three viewing directions are shown per MDS scatter plot, to display the three-dimensional nature of the plot. **a**-**c**, The first three dimensions of MDS for the digits dataset (**Fig. 2b**-**g**) show that the images are clearly grouped int four clusters. The color coding represents the ground truth label of the images, which are known, since the data is imaged separately per class in different experiments. **d**-**f**, Same as **a**-**c**, but for the letters dataset (**Fig. 2o**-**t**). **g**-**i**, Same plot, but for the experimental TUD dataset (**Fig. 3a**). The color coding of the points represents the classification result (**Fig. 3b**-**c**), since the ground truth is not known. **j**-**l**, Same plot, but for the simulated 2D NPC dataset with continuous radius variation (**Suppl. Fig. 8i**). Since the dataset does not contain discrete variations, the MDS scatter plot shows a single cloud of points. Within this cloud, the images are ordered based on their radius (see color coding), which shows that the MDS approach can clearly separate based on the variation present in the data. Subsequent clustering of the MDS space will result in clusters of images that have a similar radius.

This figure illustrates that when the dataset contains discrete variations, the MDS space shows distinct clusters that indicate to the user which value of *K* best suits the classifications. In case of a continuous variation, the MDS space will form a single cluster in which the images are sorted based on similarity. Here, any value of *K* will suffice for classification, based on the preference of the user.

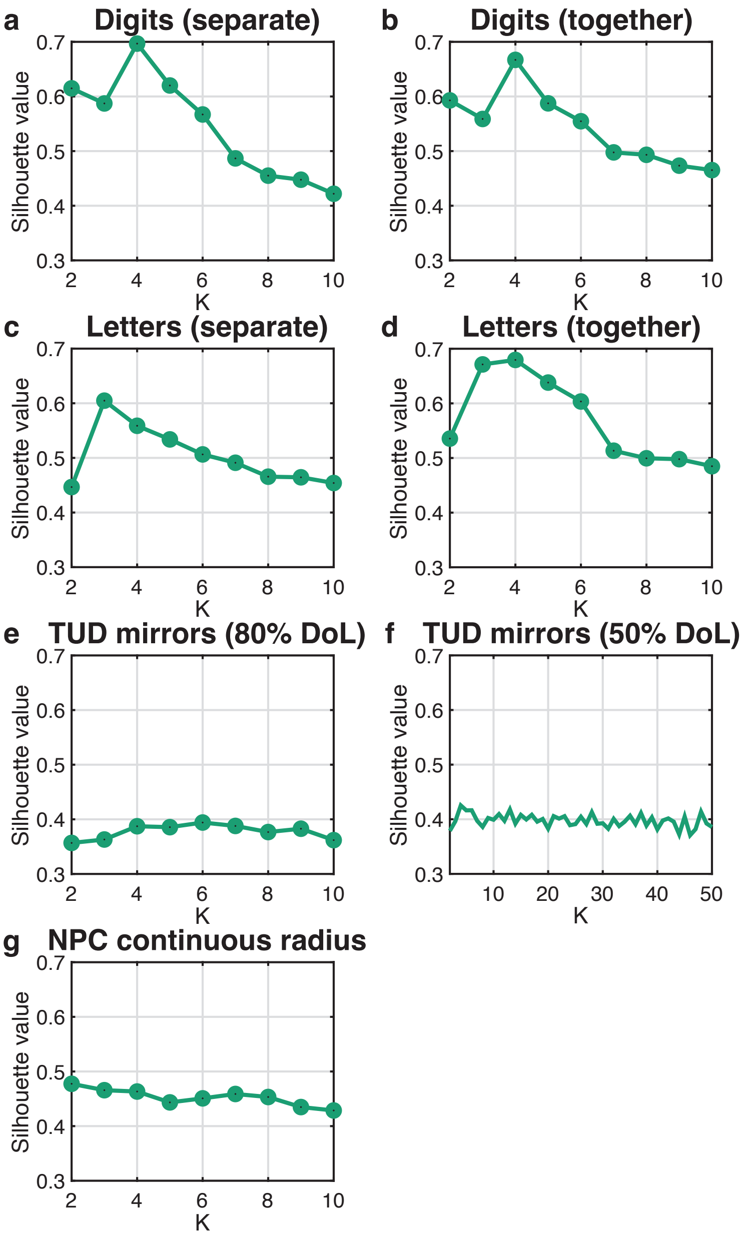

Supplementary Fig. 2 | Determine value of *K* using the silhouette cluster evaluation measure. a-**b**, The silhouette value for a range of *K*-values for the digits dataset that is either imaged separately (**Fig. 2b-g**) or together (**Fig. 2h-m**). We see that *K*=4 is giving the highest silhouette value, which is also what we can see in the MDS plot (**Suppl. Fig. 12a-c**) and what is excepted from prior knowledge about the dataset. **c**-**d**, Same plot for the letters dataset that is either imaged separately (**Fig. 2o-t**) or together (**Fig. 2u-z**). We see that *K*=3 (for separate imaging) and *K*=3/4 (for imaging together) are the best options. As explained in the main text, a value of 3 will give a decent classification performance for both datasets, but respective values of *K*=4 and *K*=5 are needed to correctly reconstruct the classes, by further classifying the misfolded particles. **e**-**f**, Same plot for the experimental TUD dataset, either with a density of labeling of 80% (**Fig. 3a**-**c**) or 50% (**Fig. 3d**-**f**). Since only a small percentage of the particles is mirrored (~2%), the silhouette plot does not give a peak at a certain *K*-value. We suggest looking at the MDS plot (**Suppl. Fig. 12**), which tells us that *K*=4 is a good value for 80% DoL. For 50% DoL, both the silhouette plot as the MDS plot do not clearly indicate a certain *K*-value, so the advice is to choose a high *K* and experiment with different values (*K*=40 turns out to be the best for this experiment). **g**, Same plot for the simulated 2D NPC dataset with continuous radius variation (**Suppl. Fig. 8i**). Since the variation in the dataset is continuous, the silhouette plot does not have a peak at a certain *K*-value. The user can pick any value of *K*, keeping in mind that a small value will give a few classes that have a lot of variation within each class, and a large value will result in reconstructions of lower quality, since each class only contains a low number of particles.

The left column here (**a**, **c**, **e** and **g**) represents the same datasets as for the MDS plots in **Suppl. Fig 12**.

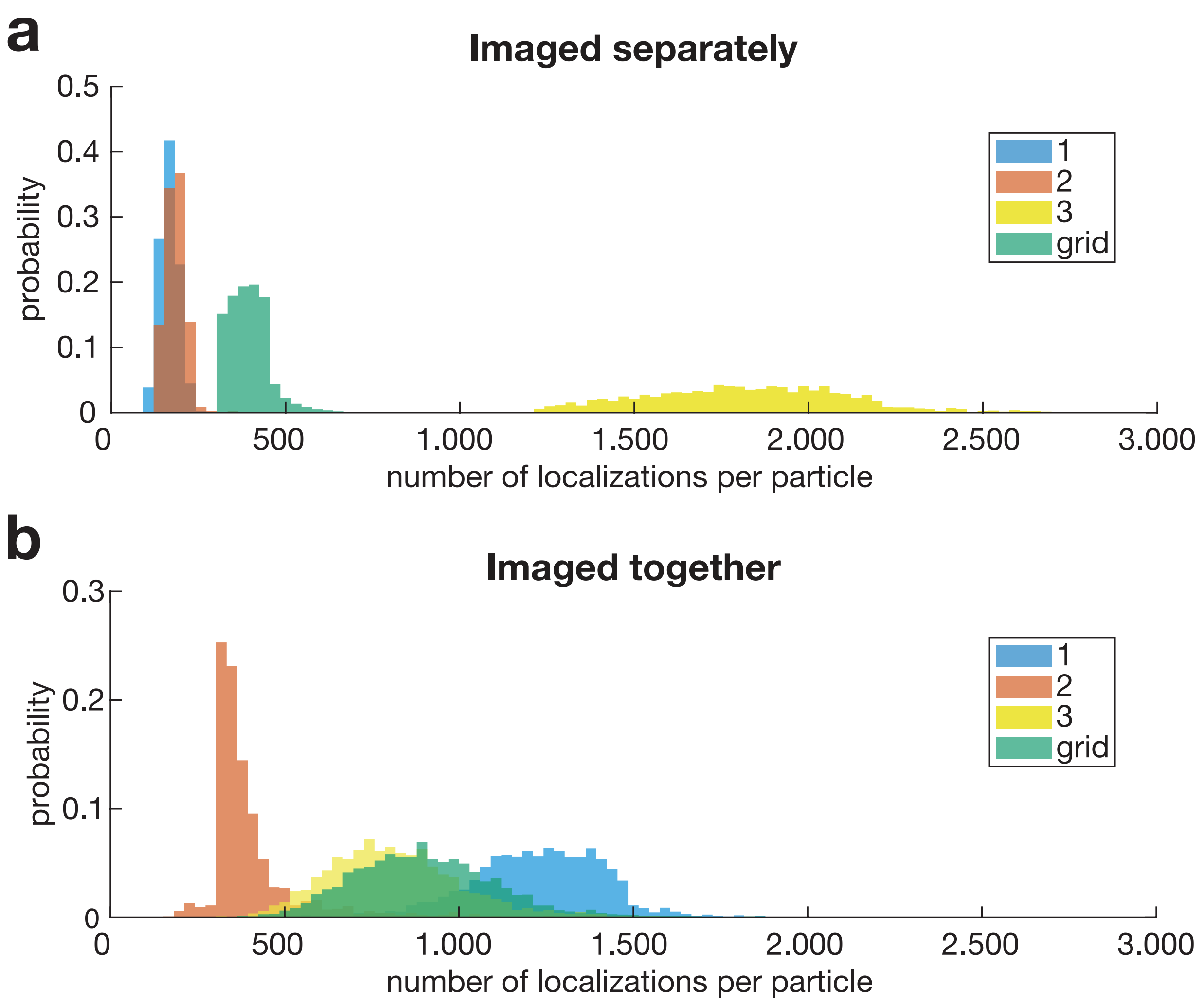

Supplementary Fig. 3 | Distributions of number of localizations per particle for digits datasets. a, Normalized distributions of number of localizations for digits 1, 2, 3 and grid that are imaged per class in separate experiments. Distributions contain 4155, 4943, 2541 and 7696 particles, respectively. **b**, Normalized distributions for digits 1, 2, 3 and grid that are imaged in one field-of-view. Distributions contain 2832, 1328, 3292 and 3653 particles, respectively. All distributions are individually normalized to have unit sum probability. We see that in **a**, digit 3 contains significantly more localizations per particle than the other structures and in **b**, digit 1 contains the most localizations. Due to this imbalance, the digit 3 and digit 1, respectively, are mostly visible in the fusion results of all particles (**Fig. 2b** and **2h**), since they are the brightest class per dataset.

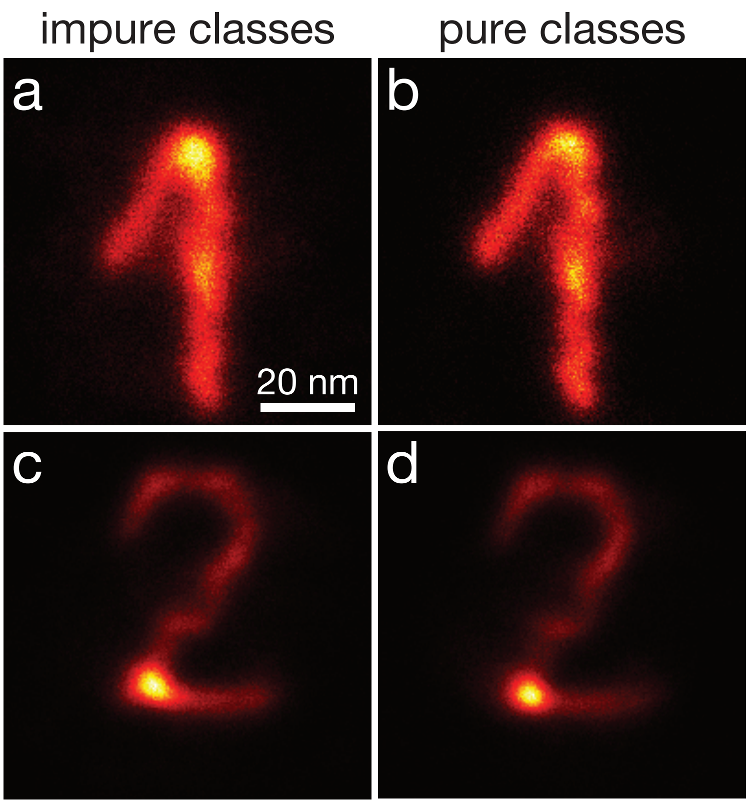

Supplementary Fig. 4 | Reconstruction of pure classes. a, Class 1 resulting from classification of 5,000 images of the digits dataset, which are imaged separately. Same image as **Fig. 2c** in the main text. As can be seen in the confusion matrix (**Fig. 2g**), this class contains 1236x digit 1, 59x digit 2, 68x digit 3 and 11x grid. **b**, Particle fusion result of only the 1236x digit 1 particles of **a**. **c**, Class 2 resulting from classification of 5,000 images of the digits dataset, which are imaged together. Same image as **Fig. 2j** in the main text. As can be seen in the confusion matrix (**Fig. 2m**), this class contains 37x digit 1, 1201x digit 2, 31x digit 3 and 40x grid. **d**, Particle fusion result of only the 1201x digit 2 particles of **c**. Scale bar of **a** applies to all.

From these particle fusion results, we can conclude that the lower quality of the reconstructions of **a** and **c** (compare to **Fig. 2d,i** of the main text) is because the data itself is of lower quality, not due to suboptimal classification performance.

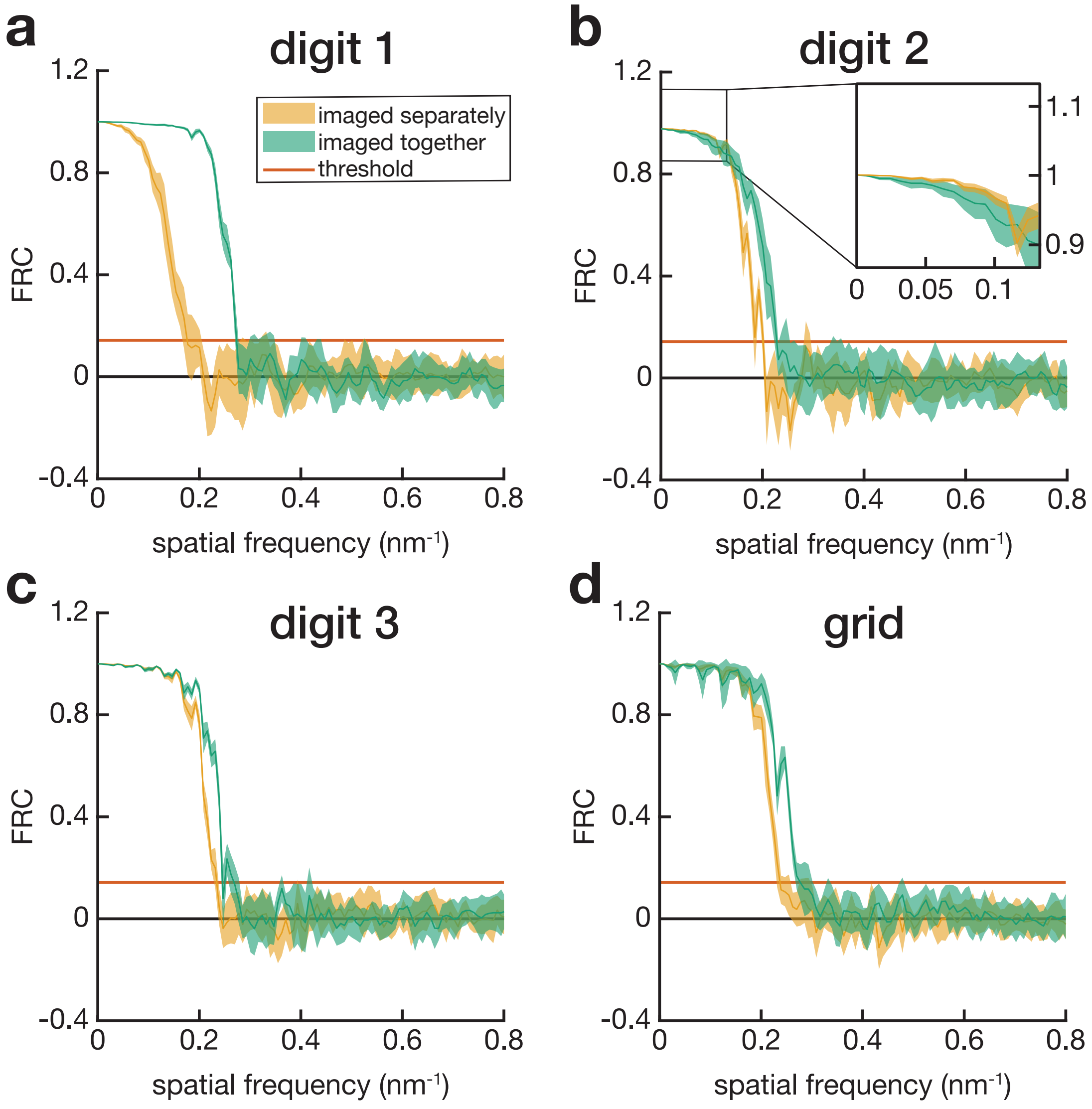

Supplementary Fig. 5 | Fourier ring correlation measurements for the digits reconstructions. a, The images classified as digit 1 (Fig. 2c and 2i) are randomly split into two equal groups, reconstructed, and FRC curves are calculated. Solid line is the mean FRC curve for 10 random splits per class and the shaded area represents one standard deviation. The FRC image resolution (crossing with 1/7 threshold) is 5.62 ± 0.39 nm for 'imaged separately' and 3.69 ± 0.02 nm for 'imaged together', defined as the average crossing of 10 random splits with one standard deviation. The significant difference in resolution is visible in the class images (**Fig. 2c** and **2i**). **b**, Same as in **a**, but for digit 2. The FRC image resolution is 5.10 ± 0.28 nm for 'imaged separately' and 4.40 ± 0.19 nm for 'imaged together'. Even though the intersection of the FRC curve with the 1/7 threshold suggests a better resolution for the 'imaged together' experiment, the images (Fig. 2d and 2j) suggest otherwise. The fact that for the low frequencies (see inset), the 'imaged separately' has a higher correlation, explains why the class image for 'image separately' shows a visually better digit 2. **c**, Same as in **a**, but for digit 3. The FRC image resolution is 4.27 ± 0.08 nm for 'imaged separately' and 3.98 ± 0.22 nm for 'imaged together'. **d**, Same as in **a**, but for the 3x4 grid structure. The FRC image resolution is 4.19 ± 0.13 nm for 'imaged separately' and 3.59 ± 0.15 nm for 'imaged together'. Legend of **a** applies to all.

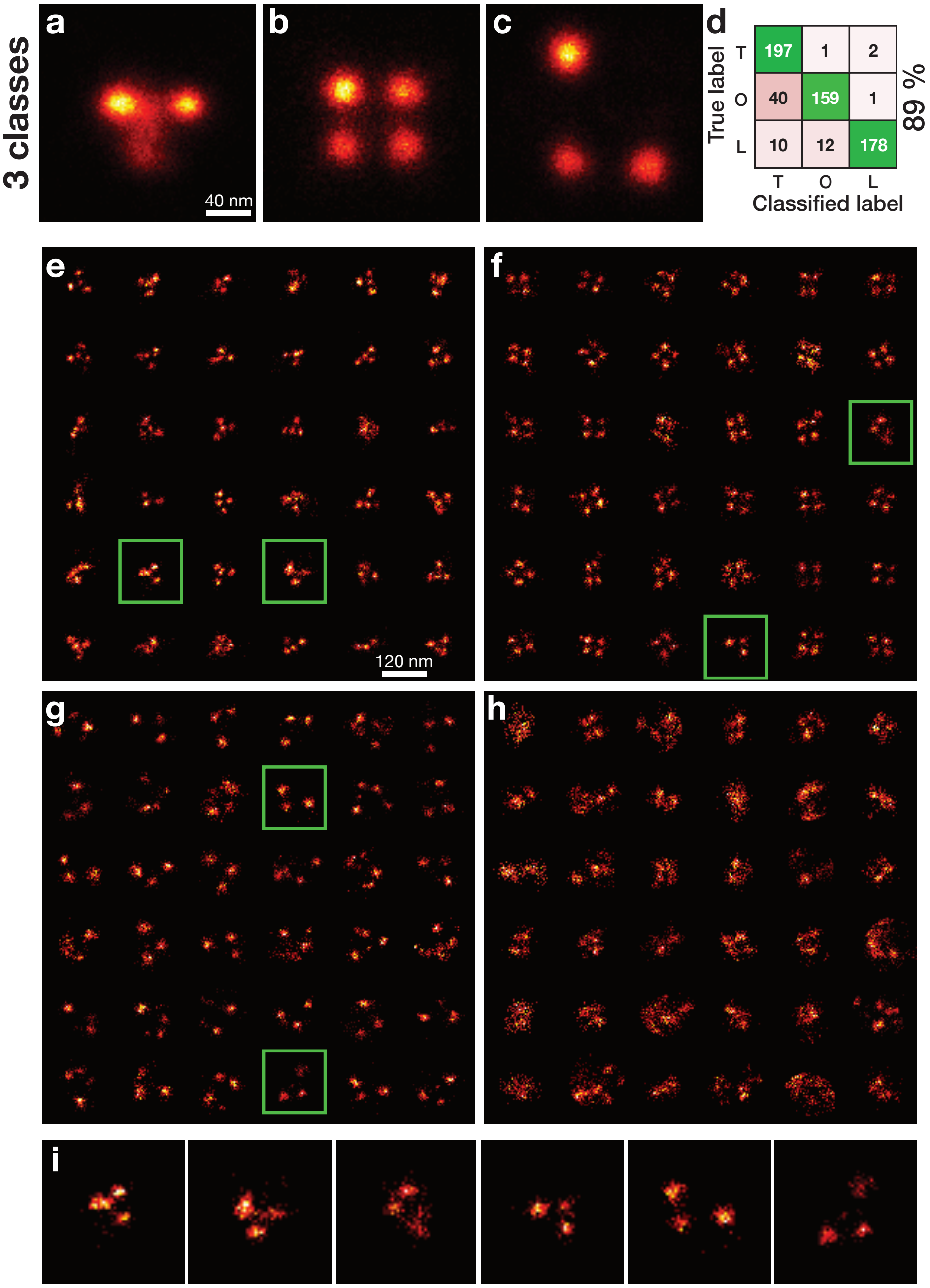

Supplementary Fig. 6 | Justification for using four classes to capture the misfolds. a-c, Classification result for 600 particles, 200 per class, of the letter dataset with separate imaging (**Fig. 2o**) are classified into three classes, containing 247, 172 and 181 particles per class, respectively. The three structures (the letters T, O and L) are clearly visible. However, the letter T is of lower quality than with classification into four classes (**Fig. 2p**). The reason is that the dataset contains misfolded structures that mixed into the other classes (as seen in the confusion matrix **d**). When classified into four classes, the misfolded images are captured in the fourth class. **d**, Confusion matrix for classification into three classes. Due to misclassification of misfolded images, the classification performance of 89% is lower than the 97.3% when a class is added to capture the misfolds (**Fig. 2t**). **e-h**, Panels show 36 representative particles per class, when the dataset is classified into four classes (**Fig. 2p-s**). It is clearly visible that **e-g** contain the letters T, O and L, where **h** contains the misfolded images. **i**, Zoom-in on the six green-outlined structures in **e-g**, all consisting of three dots, arranged in an L-shape, indicating that the TOL dataset is difficult to classify when one dots disappears due to a low density of labeling. Scale bar of **a** applies to **b**,**c** and scale bar of **e** applies to **f-h**.

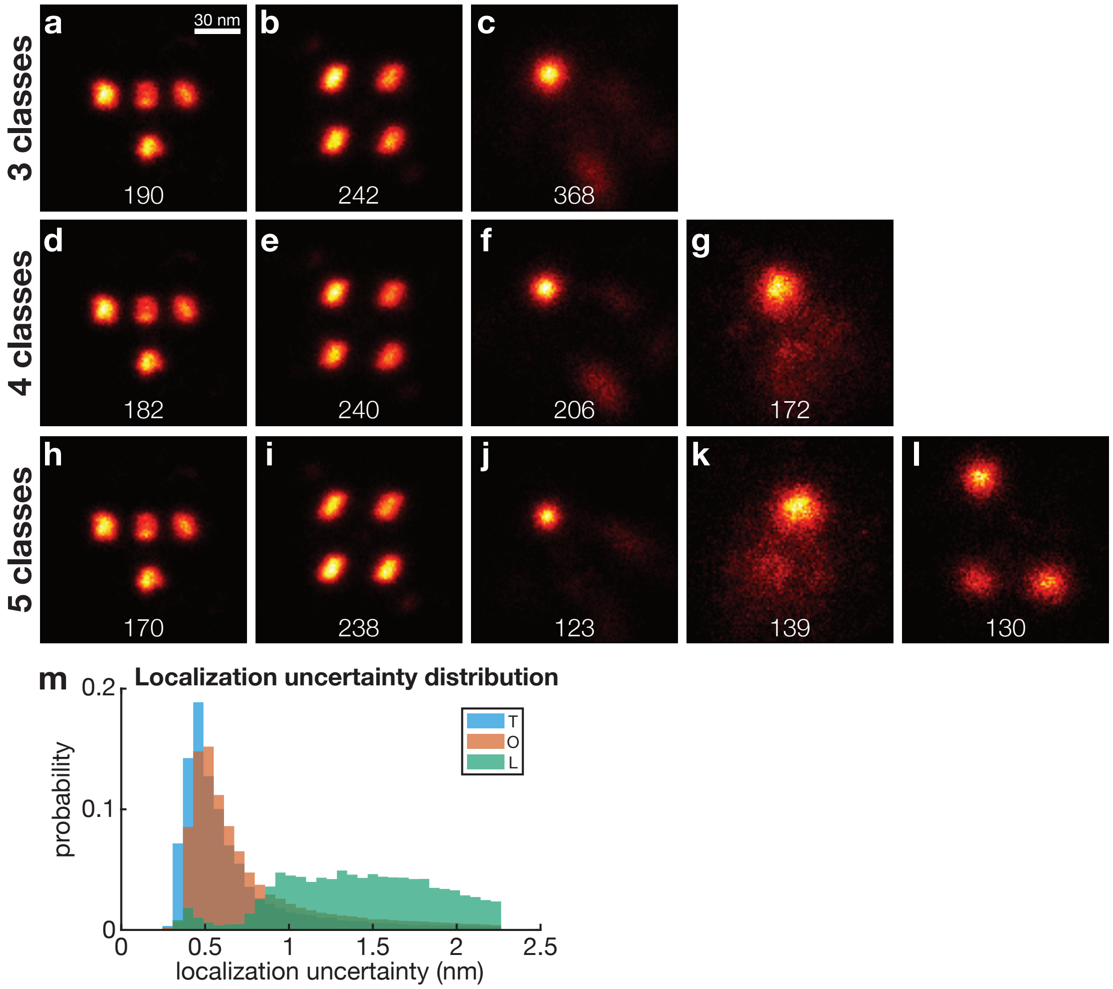

Supplementary Fig. 7 | Classification into 5 classes needed to capture the letter L. Classification of 800 particles of the letter dataset, which are imaged together (**Fig. 2u**) into: **a-c**, three classes, **d**-**g**, four classes, and **h**-**l**, five classes. The numbers below each panel indicate the number of particles contained in that class. These indicate that the "L"-structure were correctly classified when using three classes, but the reconstruction did not show the "L"-shape, due the presence of too many misfolded images, which correctly separated when using five classes. The bottom row, **h**-**l**, contains the same five images as **Fig. 2v**-**z** in the main text, but is shown here again for convenience. **m**, Distribution of localization uncertainties for the classes T (n=16659 localizations), O (n=96621 localizations) and L (n=62100 localizations) of **h**, **i** and **l**, respectively. Scale bar of **a** applies to **b**-**l**.

It is clear that classification into five classes is necessary in order to capture the letter L. One of the reasons is that the L particles have a bigger spread in localization uncertainty. This, in combination with the simpler design of only 3-4 clusters of binding sites, makes that the misfolded DNA origami structures are less well excluded in the process of particle picking, resulting in a high amount of misfolded and unrecognizable structures.

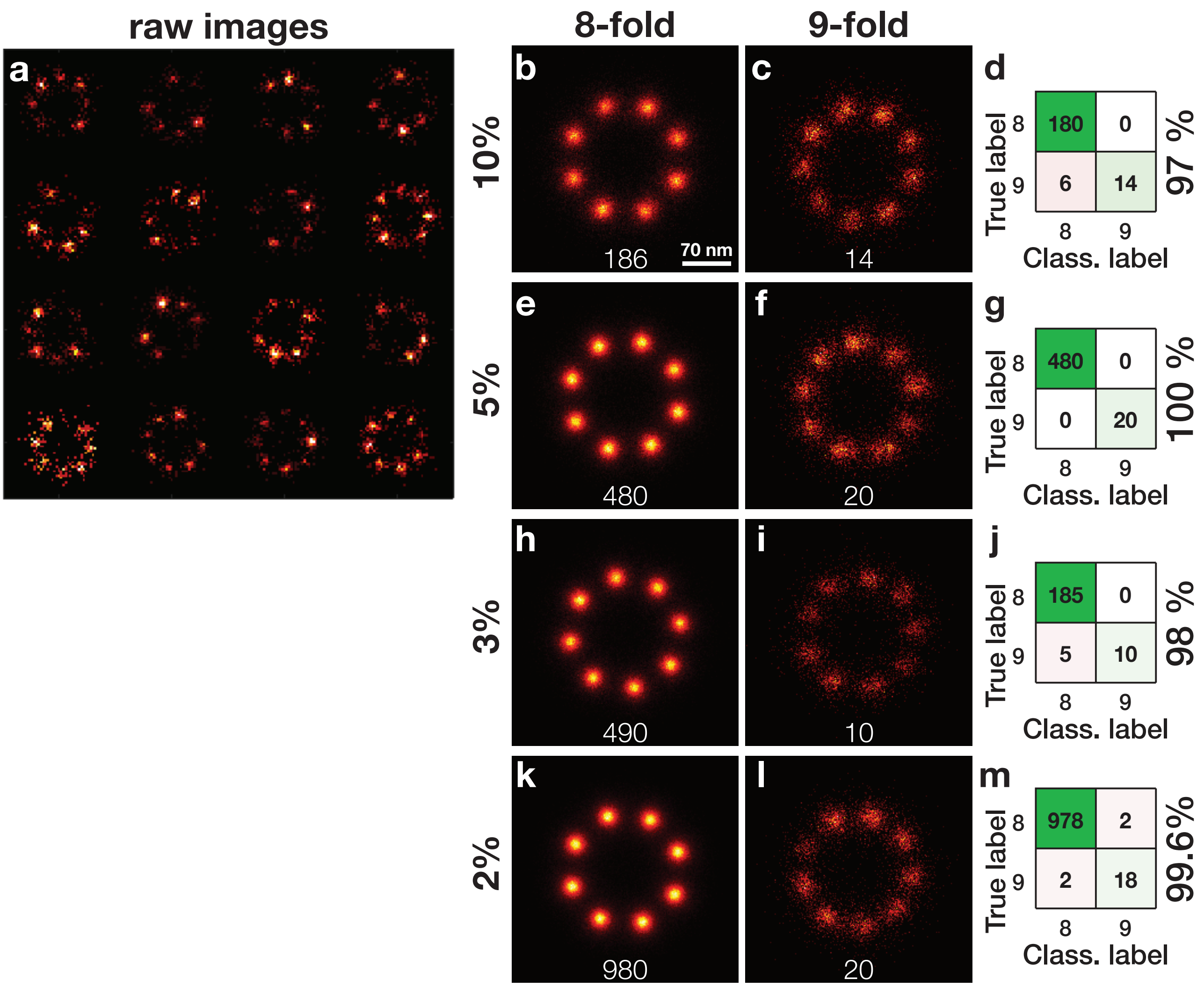

Supplementary Fig. 8 | Detecting rare classes of 9-fold simulated nuclear pores. a, 16 representative examples of simulated 8-fold symmetric rings with a diameter of 160 nm, a mean localization uncertainty of 8 nm and full labeling. The rings are simulated including a bleaching rate of 0.025 frame^{-1} as if they are imaged with STORM, and therefore have different numbers or localizations per binding site. We performed classification on four datasets containing 10, 5, 3 and 2% 9-fold symmetric rings of a total number of particles of 200, 500, 500 and 1000. The classification separates the 9-fold symmetric particles (**c**, **f**, **i**, **l**) from the 8-fold symmetric particles (**b**, **e**, **h**, **k**). Numbers below each class indicate the number of particles contained in that class, where for lower rates of 9-fold symmetric rings, larger datasets are required in order to classify them. **d**, **g**, **j**, **m,** Show classification performance, defined as the percentage of correctly classification images, and confusion matrices of the classification. For classification, the multidimensional scaling space is clustered with k-means in *K*=5, 8, 15 and 20 clusters, respectively, followed by the eigen image method to group them into two classes (*C*=2). The shown class results are reconstructed with implying the 8- and 9-fold symmetry during bootstrapping[5] for visual improvement of the class results. However, the prior symmetry knowledge is only used for the reconstruction, not for the classification itself. Scale bar of **b** applies to **c**-**i**.

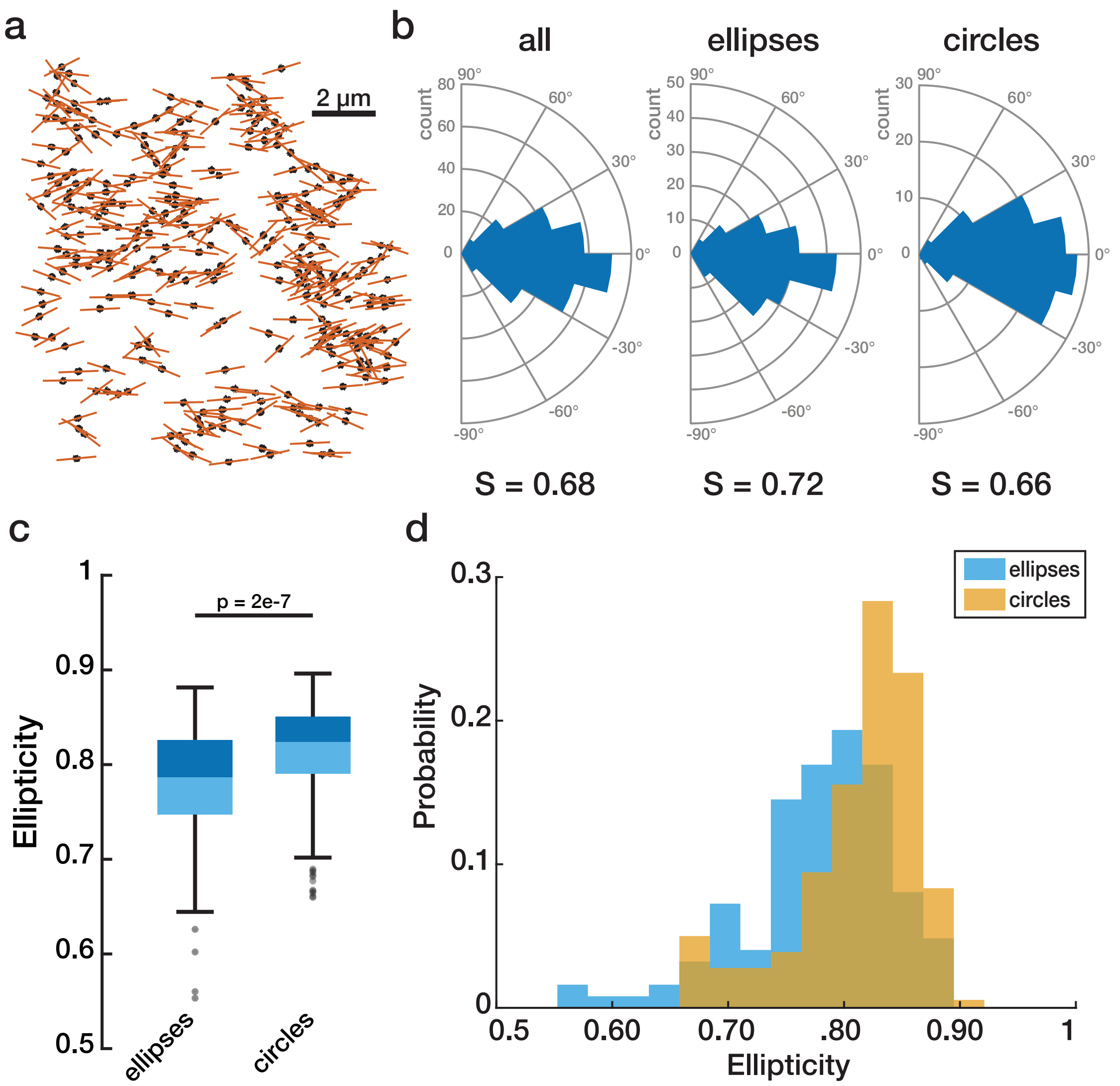

Supplementary Fig. 9 | Ellipticity of NPC integral membrane protein gp210. a, Field-of-view (FOV) showing 304 particles[3], with the orientation of the major axis of fitted ellipses for every particle shown in orange. **b**, Polar histograms for the orientation of the major axes of the elliptical fits of **a**. First histogram contains all 304 particles. Second and third histogram contain the particles for the elliptical and circular class, with 180 and 124 particles, respectively. The particles are classified into four classes, of which two resulted in ellipses and two in circles, grouped here per shape. The order parameter values (parameter *S*, see Methods) show that the particles classified as ellipses are mutually more aligned than the particles classified as circles. **c**, Boxplots of the ellipticity values (see Methods) for the elliptical (n=124) and circular (n=180) classes. Two-sided Kolmogorov-Smirnov statistic shows that the distributions are significantly different with a p-value of 2.28×10^{-7}. In each box, the central line marks the median, the box edges indicate the 25th and 75th percentiles, respectively. The whiskers extend to the most extreme data points not considered outliers, and the outliers are plotted as dots outside the whisker range. **d**, Same distributions as in **c**, but shown as histograms.

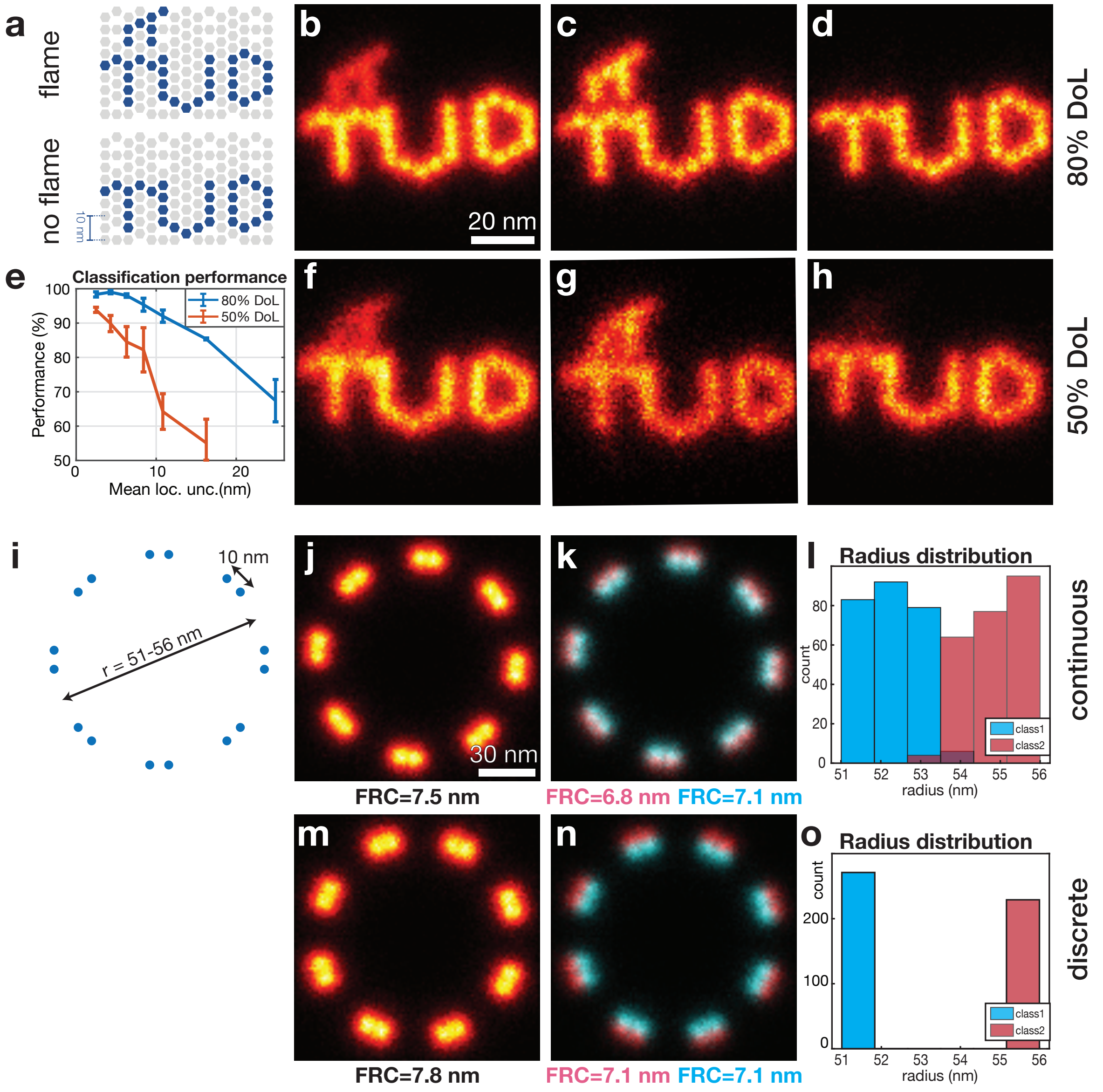

Supplementary Fig. 10 | Classification of multiple simulated datasets. a, Templates of two DNA origami structures used for simulating the particles, TUD-logos with and without flame. **b**, Fusion result of 200 particles (100 with and 100 without flame), 80% DoL, mean localization uncertainty of 4.3 nm and simulated with PAINT. **c**-**d**, Classification result of **b**, containing 98 and 102 particles, respectively. **e**, Classification performance over mean localization accuracy for different labeling densities (200 particles per experiment, equally divided over the two classes). Classification performance is represented as the mean of n=3 independent datasets, error bars represent one standard deviation. **f**-**h**, Same as **b**-**d**, but with 50% DoL. **g** contains 78 particles and **h** 122. **i**, Model used to simulate the NPC particles. Ring with 8 doublets where the two emitters per doublet are 10 nm apart. **j**, Fusion result of 500 particles, simulated with a uniformly distributed radius in the range 51-56 nm, mean localization uncertainty of 6.3 nm, 70% DoL and simulated with PAINT. **k**, Two-color overlay of the classification result of **j**, containing 260 particles in class 1 (cyan) and 240 particles in class 2 (red). The overlay of red and cyan displays white. **l**, Radius distribution per class. Radii are ground-truth radii used in simulating the particles. **m**-**o**, Same as **j**-**l**, but with two discrete radii of 51 and 56 nm. For the average and the individual classes, the FRC values are mentioned below the figures. The mentioned values are the mean 1/7-crossing resolutions of 50 random splits. Scale bar in **b** applies to **c**-**d** and **f**-**h**. Scale bar in **j** applies to **k**, **m**-**n**.

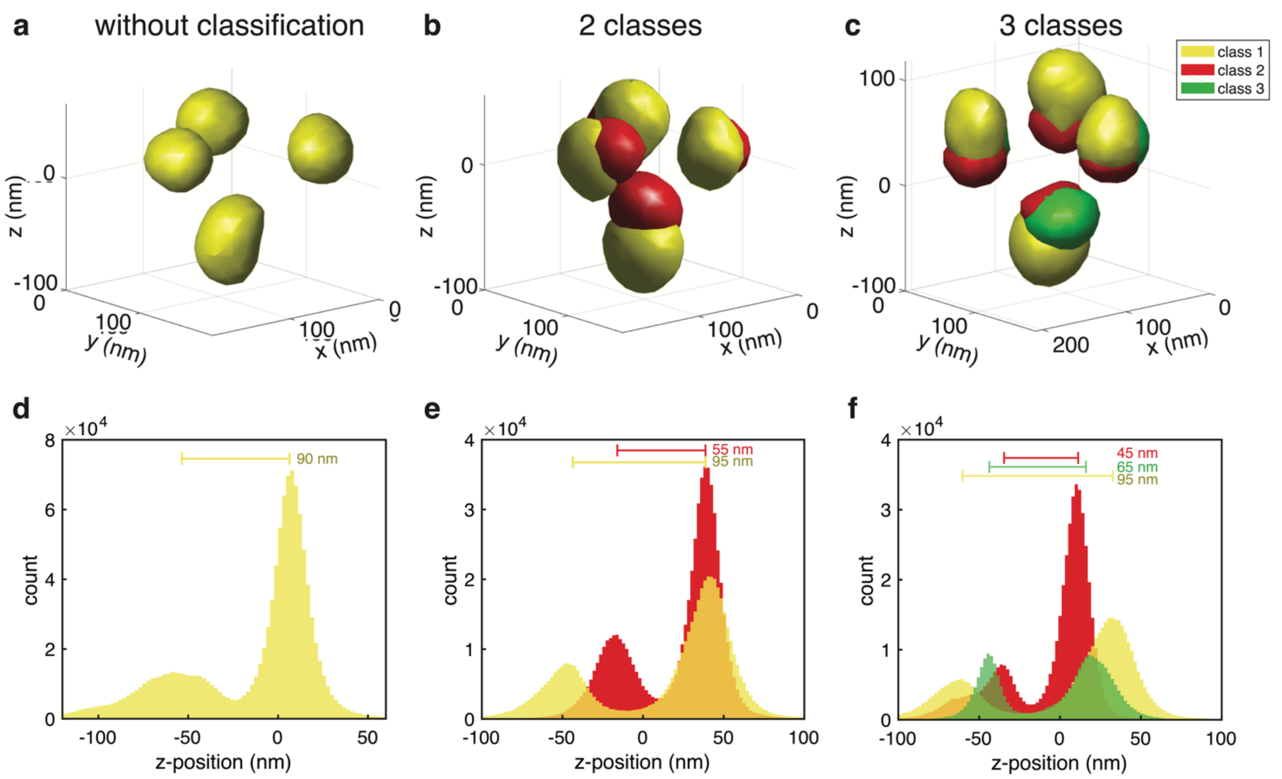

Supplementary Fig. 11 | Classification on 3D DNA-origami tetrahedron structure reveals variation in height due to folding variability. a, Surface plot visualization of the fusion result of 218 tetrahedron particles (see Methods), which in total contain 1,131,181 localizations. The elongated lower blob indicates that the individual structures vary in height. **b**, Classification result into two classes (*K*=2), where the classes contain 96 and 122 particles, respectively. **c**, Classification result into three classes (*K*=3), where the classes contain 74, 95 and 49 particles, respectively. **d**-**f**, Distributions of the z-positions of the localizations for the classes shown in **a**-**c**. The distributions show clearly that the classification algorithm separates the particles based on the height difference between the upper triangle and the bottom vertex. In **b**,**c**, the classes are individually reconstructed and afterwards aligned to each other. Estimated mean localization uncertainty in the z-direction is 8 nm.

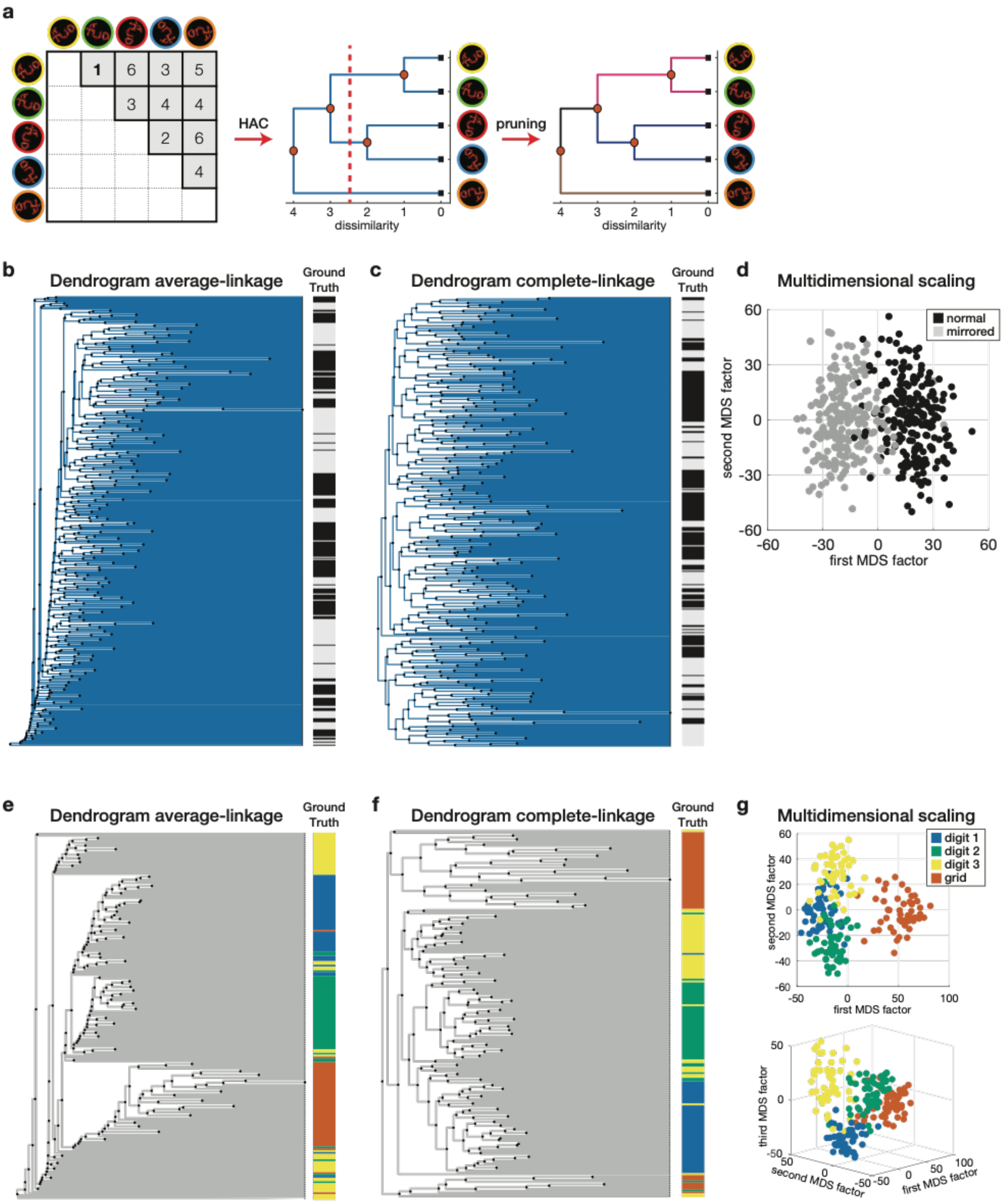

Supplementary Fig. 12 | Comparison between the MDS and HAC approach. a, Schematic representation of the hierarchical agglomerative clustering approach. The upper-triangular matrix of dissimilarity values is used to construct to construct the dendrogram using hierarchical agglomerative clustering (HAC), with the single-linkage distance criterion in this example. The obtained dendrogram is pruned at a certain threshold (red-dotted line) resulting in a number of distinct clusters (in this case three) that are reconstructed per cluster. **b**, HAC dendrogram with average-linkage criterion for 440 experimental TUD-logos with 50% DoL, of which 220 are manually flipped. The gray-scale bar on right-side of the dendrogram indicates the ground truth label for the particles, for legend see **d**. **c**, Same as **b**, but for the complete-linkage criterion. **d**, Scatterplot showing the first two dimensions of the MDS embedding. The gray-scale colors indicate the ground truth class labels. **e**-**g**, Same as **b**-**d**, but for 200 particles of the digits dataset (50 per class), that are imaged separately. The colorbars on the right-side of the dendrograms indicate the ground truth label for the particles, for legend see **g**. **g**, (top) The scatterplot showing the first two dimensions of the MDS embedding, (bottom) scatterplot showing the first three dimensions of the MDS embedding. Multiple views are shown to get a better idea about the three-dimensional configuration of the particles. The colors indicate the ground truth class labels.

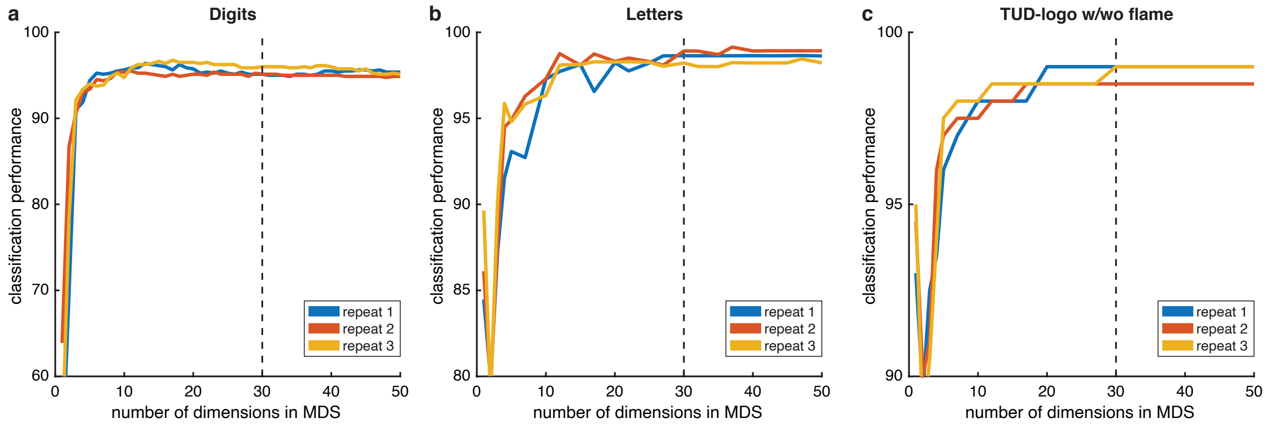

Supplementary Fig. 13 | Optimal number of dimensions in multidimensional scaling. a, Classification performance for different numbers of dimensions in MDS for 800 particles (200 per class) of the digits dataset which is imaged separately. **b**, Classification performance for different numbers of dimensions in MDS for 600 particles (200 per class) of the letters dataset which is imaged separately. **c**, Classification performance for different numbers of dimensions in MDS for 200 particles (100 per class) of the simulated TUD-logos with a mean localization uncertainty of 4.3 nm and 80% DoL. Half of the particles does not have the flame above the first letter. Three colors in all plots represent independent repeats on different datasets.

We can conclude that the optimal classification performance is reached around 15 dimensions. We take as a rule of thumb twice this value and therefore embed the particles into 30 dimensions in all reported experiments in this paper.

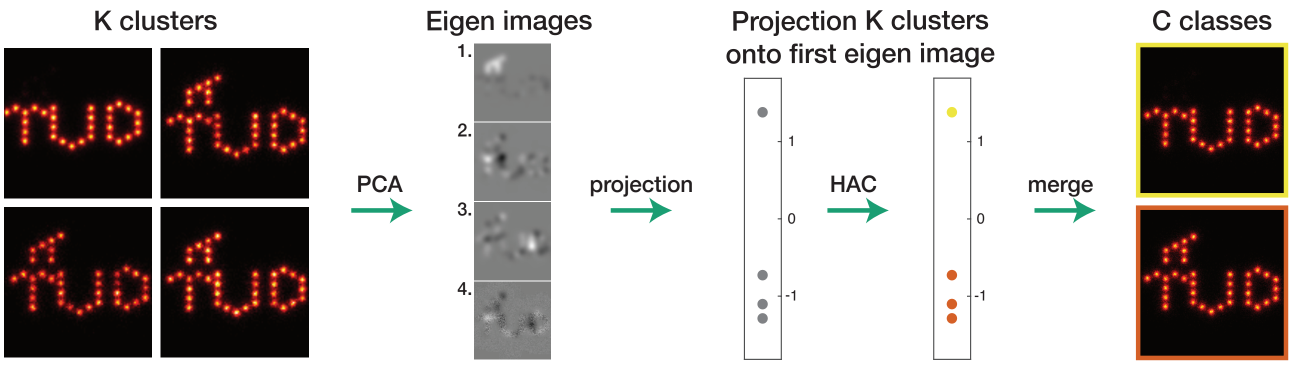

Supplementary Fig. 14 | Optional further classification for datasets with small subgroups of structurally different particles. After k-means clustering the multidimensional scaling space into *K* clusters (**Fig. 1**), principal component analysis (PCA) on the *K* reconstructions gives *K* eigen images. All clusters are projected onto the first eigen image and the resulting weights are grouped into *C* classes by hierarchical agglomerative clustering (HAC). The grouped clusters are merged into *C* classes, with $C<K$.

Supplementary Tab. 1 | M13mp18 p7249 sequence

TTCCCTTCCTTTCTCGCCACGTTCGCCGGCTTTCCCCGTCAAGCTCTAAATCGGGGGCTCCCTTTAGGGTTCCGATTTAGTGCTTTACGGCACCTCGACCCCAAAAA
ACTTGATTTGGGTGATGGTTCACGTAGTGGGCCATCGCCCTGATAGACGGTTTTTCGCCCTTTGACGTTGGAGTCCACGTTCTTTAATAGTGGACTCTTGTTCCAA
ACTGGAACAACACTCAACCCTATCTCGGGCTATTCTTTTGATTTATAAGGGATTTTGCCGATTTCGGAACCACCATCAAACAGGATTTTCGCCTGCTGGGGCAAAC
CAGCGTGGACCGCTTGCTGCAACTCTCTCAGGGCCAGGCGGTGAAGGGCAATCAGCTGTTGCCCGTCTCACTGGTGAAAAGAAAAACCACCCTGGCGCCCAATA
CGCAAACCGCCTCTCCCCGCGCGTTGGCCGATTCATTAATGCAGCTGGCACGACAGGTTTCCCGACTGGAAAGCGGGCAGTGAGCGCAACGCAATTAATGTGAG
TTAGCTCACTCATTAGGCACCCCAGGCTTTACACTTTATGCTTCCGGCTCGTATGTTGTGTGGAATTGTGAGCGGATAACAATTTCACACAGGAAACAGCTATGAC
CATGATTACGAATTCGAGCTCGGTACCCGGGGATCCTCTAGAGTCGACCTGCAGGCATGCAAGCTTGGCACTGGCCGTCGTTTTACAACGTCGTGACTGGGAAA
ACCCTGGCGTTACCCAACTTAATCGCCTTGCAGCACATCCCCCTTTCGCCAGCTGGCGTAATAGCGAAGAGGCCCGCACCGATCGCCCTTCCCAACAGTTGCGCA
GCCTGAATGGCGAATGGCGCTTTGCCTGGTTTCCGGCACCAGAAGCGGTGCCGGAAAGCTGGCTGGAGTGCGATCTTCCTGAGGCCGATACTGTCGTCGTCCCC
TCAAACTGGCAGATGCACGGTTACGATGCGCCCATCTACACCAACGTGACCTATCCCATTACGGTCAATCCGCCGTTTGTTCCCACGGAGAATCCGACGGGTTGTT
ACTCGCTCACATTTAATGTTGATGAAAGCTGGCTACAGGAAGGCCAGACGCGAATTATTTTTGATGGCGTTCCTATTGGTTAAAAAATGAGCTGATTTAACAAAA
ATTTAATGCGAATTTTAACAAAATATTAACGTTTACAATTTAAATATTTGCTTATACAATCTTCCTGTTTTTGGGGCTTTTCTGATTATCAACCGGGGTACATATGAT
TGACATGCTAGTTTTACGATTACCGTTCATCGATTCTCTTGTTTGCTCCAGACTCTCAGGCAATGACCTGATAGCCTTTGTAGATCTCTCAAAAATAGCTACCCTCTC
CGGCATTAATTTATCAGCTAGAACGGTTGAATATCATATTGATGGTGATTTGACTGTCTCCGGCCTTTCTCACCCTTTTGAATCTTTACCTACACATTACTCAGGCAT
TGCATTTAAAATATATGAGGGTTCTAAAAATTTTTATCCTTGCGTTGAAATAAAGGCTTCTCCCGCAAAAGTATTACAGGGTCATAATGTTTTTGGTACAACCGATT
TAGCTTTATGCTCTGAGGCTTTATTGCTTAATTTTGCTAATTCTTTGCCTTGCCTGTATGATTTATTGGATGTTAATGCTACTACTATTAGTAGAATTGATGCCACCTT
TTCAGCTCGCGCCCCAAATGAAAATATAGCTAAACAGGTTATTGACCATTTGCGAAATGTATCTAATGGTCAAACTAAATCTACTCGTTCGCAGAATTGGGAATCA
ACTGTTATATGGAATGAAACTTCCAGACACCGTACTTTAGTTGCATATTTAAAACATGTTGAGCTACAGCATTATATTCAGCAATTAAGCTCTAAGCCATCCGCAA
AAATGACCTCTTATCAAAAGGAGCAATTAAAGGTACTCTCTAATCCTGACCTGTTGGAGTTTGCTTCCGGTCTGGTTCGCTTTGAAGCTCGAATTAAAACGCGATA
TTTGAAGTCTTTCGGGCTTCCTCTTAATCTTTTTGATGCAATCCGCTTTGCTTCTGACTATAATAGTCAGGGTAAAGACCTGATTTTTGATTTATGGTCATTCTCGTTT
TCTGAACTGTTTAAAGCATTTGAGGGGGATTCAATGAATATTTATGACGATTCCGCAGTATTGGACGCTATCCAGTCTAAACATTTTACTATTACCCCCTCTGGCAA
AACTTCTTTTGCAAAAGCCTCTCGCTATTTTGGTTTTTATCGTCGTCTGGTAAACGAGGGTTATGATAGTGTTGCTCTTACTATGCCTCGTAATTCCTTTTGGCGTTA
TGTATCTGCATTAGTTGAATGTGGTATTCCTAAATCTCAACTGATGAATCTTTCTACCTGTAATAATGTTGTTCCGTTAGTTCGTTTTATTAACGTAGATTTTTCTTCC
CAACGTCCTGACTGGTATAATGAGCCAGTTCTTAAAATCGCATAAGGTAATTCACAATGATTAAAGTTGAAATTAAACCATCTCAAGCCCAATTTACTACTCGTTCT
GGTGTTTCTCGTCAGGGCAAGCCTTATTCACTGAATGAGCAGCTTTGTTACGTTGATTTGGGTAATGAATATCCGGTTCTTGTCAAGATTACTCTTGATGAAGGTC
AGCCAGCCTATGCGCCTGGTCTGTACACCGTTCATCTGTCCTCTTTCAAAGTTGGTCAGTTCGGTTCCCTTATGATTGACCGTCTGCGCCTCGTTCCGGCTAAGTAA
CATGGAGCAGGTCGCGGATTTCGACACAATTTATCAGGCGATGATACAAATCTCCGTTGTACTTTGTTTCGCGCTTGGTATAATCGCTGGGGGTCAAAGATGAGT
GTTTTAGTGTATTCTTTTGCCTCTTTCGTTTTAGGTTGGTGCCTTCGTAGTGGCATTACGTATTTTACCCGTTTAATGGAAACTTCCTCATGAAAAAGTCTTTAGTCC
TCAAAGCCTCTGTAGCCGTTGCTACCCTCGTTCCGATGCTGTCTTTCGCTGCTGAGGGTGACGATCCCGCAAAAGCGGCCTTTAACTCCCTGCAAGCCTCAGCGAC
CGAATATATCGGTTATGCGTGGGCGATGGTTGTTGTCATTGTCGGCGCAACTATCGGTATCAAGCTGTTTAAGAAATTCACCTCGAAAGCAAGCTGATAAACCGA
TACAATTAAAGGCTCCTTTTGGAGCCTTTTTTTTGGAGATTTTCAACGTGAAAAAATTATTATTCGCAATTCCTTTAGTTGTTCCTTTCTATTCTCACTCCGCTGAAAC
TGTTGAAAGTTGTTTAGCAAAATCCCATACAGAAAATTCATTTACTAACGTCTGGAAAGACGACAAAACTTTAGATCGTTACGCTAACTATGAGGGCTGTCTGTG
GAATGCTACAGGCGTTGTAGTTTGTACTGGTGACGAAACTCAGTGTTACGGTACATGGGTTCCTATTGGGCTTGCTATCCCTGAAAATGAGGGTGGTGGCTCTGA
GGGTGGCGGTTCTGAGGGTGGCGGTTCTGAGGGTGGCGGTACTAAACCTCCTGAGTACGGTGATACACCTATTCCGGGCTATACTTATATCAACCCTCTCGACG
GCACTTATCCGCCTGGTACTGAGCAAAACCCCGCTAATCCTAATCCTTCTCTTGAGGAGTCTCAGCCTCTTAATACTTTCATGTTTCAGAATAATAGGTTCCGAAAT
AGGCAGGGGGCATTAACTGTTTATACGGGCACTGTTACTCAAGGCACTGACCCCGTTAAAACTTATTACCAGTACACTCCTGTATCATCAAAAGCCATGTATGAC
GCTTACTGGAACGGTAAATTCAGAGACTGCGCTTTCCATTCTGGCTTTAATGAGGATTTATTTGTTTGTGAATATCAAGGCCAATCGTCTGACCTGCCTCAACCTCC
TGTCAATGCTGGCGGCGGCTCTGGTGGTGGTTCTGGTGGCGGCTCTGAGGGTGGTGGCTCTGAGGGTGGCGGTTCTGAGGGTGGCGGCTCTGAGGGAGGCGG
TTCCGGTGGTGGCTCTGGTTCCGGTGATTTTGATTATGAAAAGATGGCAAACGCTAATAAGGGGGCTATGACCGAAAATGCCGATGAAAACGCGCTACAGTCTG
ACGCTAAAGGCAAACTTGATTCTGTCGCTACTGATTACGGTGCTGCTATCGATGGTTTCATTGGTGACGTTTCCGGCCTTGCTAATGGTAATGGTGCTACTGGTGA
TTTTGCTGGCTCTAATTCCCAAATGGCTCAAGTCGGTGACGGTGATAATTCACCTTTAATGAATAATTTCCGTCAATATTTACCTTCCCTCCCTCAATCGGTTGAATG
TCGCCCTTTTGTCTTTGGCGCTGGTAAACCATATGAATTTTCTATTGATTGTGACAAAATAAACTTATTCCGTGGTGTCTTTGCGTTTCTTTTATATGTTGCCACCTTT
ATGTATGTATTTTCTACGTTTGCTAACATACTGCGTAATAAGGAGTCTTAATCATGCCAGTTCTTTTGGGTATTCCGTTATTATTGCGTTTCCTCGGTTTCCTTCTGG
TAACTTTGTTCGGCTATCTGCTTACTTTTCTTAAAAAGGGCTTCGGTAAGATAGCTATTGCTATTTCATTGTTTCTTGCTCTTATTATTGGGCTTAACTCAATTCTTGT
GGGTTATCTCTCTGATATTAGCGCTCAATTACCCTCTGACTTTGTTCAGGGTGTTCAGTTAATTCTCCCGTCTAATGCGCTTCCCTGTTTTTATGTTATTCTCTCTGTA
AAGGCTGCTATTTTCATTTTTGACGTTAAACAAAAAATCGTTTCTTATTTGGATTGGGATAAATAATATGGCTGTTTATTTTGTAACTGGCAAATTAGGCTCTGGAA
AGACGCTCGTTAGCGTTGGTAAGATTCAGGATAAAATTGTAGCTGGGTGCAAAATAGCAACTAATCTTGATTTAAGGCTTCAAAACCTCCCGCAAGTCGGGAGG
TTCGCTAAAACGCCTCGCGTTCTTAGAATACCGGATAAGCCTTCTATATCTGATTTGCTTGCTATTGGGCGCGGTAATGATTCCTACGATGAAAATAAAAACGGCT
TGCTTGTTCTCGATGAGTGCGGTACTTGGTTTAATACCCGTTCTTGGAATGATAAGGAAAGACAGCCGATTATTGATTGGTTTCTACATGCTCGTAAATTAGGATG
GGATATTATTTTTCTTGTTCAGGACTTATCTATTGTTGATAAACAGGCGCGTTCTGCATTAGCTGAACATGTTGTTTATTGTCGTCGTCTGGACAGAATTACTTTACC
TTTTGTCGGTACTTTATATTCTCTTATTACTGGCTCGAAAATGCCTCTGCCTAAATTACATGTTGGCGTTGTTAAATATGGCGATTCTCAATTAAGCCCTACTGTTGA

GCGTTGGCTTTATACTGGTAAGAATTTGTATAACGCATATGATACTAAACAGGCTTTTTCTAGTAATTATGATTCCGGTGTTTATTCTTATTTAACGCCTTATTTATC
ACACGGTCGGTATTTCAAACCATTAAATTTAGGTCAGAAGATGAAATTAACTAAAATATATTTGAAAAAGTTTTCTCGCGTTCTTTGTCTTGCGATTGGATTTGCAT
CAGCATTTACATATAGTTATATAACCCAACCTAAGCCGGAGGTTAAAAAGGTAGTCTCTCAGACCTATGATTTTGATAAATTCACTATTGACTCTTCTCAGCGTCTT
AATCTAAGCTATCGCTATGTTTTCAAGGATTCTAAGGGAAAATTAATTAATAGCGACGATTTACAGAAGCAAGGTTATTCACTCACATATATTGATTTATGTACTGT
TTCCATTAAAAAAGGTAATTCAAATGAAATTGTTAAATGTAATTAATTTTGTTTTCTTGATGTTTGTTTCATCATCTTCTTTTGCTCAGGTAATTGAAATGAATAATT
CGCCTCTGCGCGATTTTGTAACTTGGTATTCAAAGCAATCAGGCGAATCCGTTATTGTTTCTCCCGATGTAAAAGGTACTGTTACTGTATATTCATCTGACGTTAAA
CCTGAAAATCTACGCAATTTCTTTATTTCTGTTTTACGTGCAAATAATTTTGATATGGTAGGTTCTAACCCTTCCATTATTCAGAAGTATAATCCAAACAATCAGGAT
TATATTGATGAATTGCCATCATCTGATAATCAGGAATATGATGATAATTCCGCTCCTTCTGGTGGTTTCTTTGTTCCGCAAAATGATAATGTTACTCAAACTTTTAAA
ATTAATAACGTTCGGGCAAAGGATTTAATACGAGTTGTCGAATTGTTTGTAAAGTCTAATACTTCTAAATCCTCAAATGTATTATCTATTGACGGCTCTAATCTATT
AGTTGTTAGTGCTCCTAAAGATATTTTAGATAACCTTCCTCAATTCCTTTCAACTGTTGATTTGCCAACTGACCAGATATTGATTGAGGGTTTGATATTTGAGGTTC
AGCAAGGTGATGCTTTAGATTTTTCATTTGCTGCTGGCTCTCAGCGTGGCACTGTTGCAGGCGGTGTTAATACTGACCGCCTCACCTCTGTTTTATCTTCTGCTGGT
GGTTCGTTCGGTATTTTTAATGGCGATGTTTTAGGGCTATCAGTTCGCGCATTAAAGACTAATAGCCATTCAAAAATATTGTCTGTGCCACGTATTCTTACGCTTTC
AGGTCAGAAGGGTTCTATCTCTGTTGGCCAGAATGTCCCTTTTATTACTGGTCGTGTGACTGGTGAATCTGCCAATGTAAATAATCCATTTCAGACGATTGAGCGT
CAAAATGTAGGTATTTCCATGAGCGTTTTTCCTGTTGCAATGGCTGGCGGTAATATTGTTCTGGATATTACCAGCAAGGCCGATAGTTTG

Supplementary Tab. 2 | Rectangular DNA origami staple strands

Plate	Pos	Name	Sequence	Letter O	Letter T	Letter L
1	A1	21[32]23[31]BLK	TTTTCACTCAAAGGGCGAAAAACCATCACC			P7
1	A2	19[32]21[31]BLK	GTCGACTTCGGCCAACGCGCGGGGTTTTTC			P7
1	A3	17[32]19[31]BLK	TGCATCTTTCCCAGTCACGACGGCCTGCAG			P7
1	A4	15[32]17[31]BLK	TAATCAGCGGATTGACCGTAATCGTAACCG			
1	A5	13[32]15[31]BLK	AACGCAAAATCGATGAACGGTACCGGTTGA			
1	A6	11[32]13[31]BLK	AACAGTTTTGTACCAAAAACATTTTATTTC			
1	A7	9[32]11[31]BLK	TTTACCCCAACATGTTTTAAATTTCCATAT		P3	
1	A8	7[32]9[31]BLK	TTTAGGACAAATGCTTTAAACAATCAGGTC			
1	A9	5[32]7[31]BLK	CATCAAGTAAAACGAACTAACGAGTTGAGA			
1	A10	3[32]5[31]BLK	AATACGTTTGAAAGAGGACAGACTGACCTT			
1	A11	1[32]3[31]BLK	AGGCTCCAGAGGCTTTGAGGACACGGGTAA			P7
1	A12	0[47]1[31]BLK	AGAAAGGAACAACTAAAGGAATTCAAAAAAA			P7
1	B1	23[32]22[48]BLK	CAAATCAAGTTTTTTGGGGTCGAAACGTGGA			P7
1	B2	22[47]20[48]BLK	CTCCAACGCAGTGAGACGGGCAACCAGCTGCA			P7
1	B3	20[47]18[48]BLK	TTAATGAACTAGAGGATCCCCGGGGGGTAACG			
1	B4	18[47]16[48]BLK	CCAGGGTTGCCAGTTTGAGGGGACCCGTGGGA			
1	B5	16[47]14[48]BLK	ACAAACGGAAAAGCCCCAAAAACACTGGAGCA		P3	
1	B6	14[47]12[48]BLK	AACAAGAGGGATAAAAATTTTTAGCATAAAGC		P3	
1	B7	12[47]10[48]BLK	TAAATCGGGATTCCCAATTCTGCGATATAATG		P3	
1	B8	10[47]8[48]BLK	CTGTAGCTTGACTATTATAGTCAGTTCATTGA			
1	B9	8[47]6[48]BLK	ATCCCCCTATACCACATTCAACTAGAAAAATC			
1	B10	6[47]4[48]BLK	TACGTTAAAGTAATCTTGACAAGAACCGAACT			P7
1	B11	4[47]2[48]BLK	GACCAACTAATGCCACTACGAAGGGGGTAGCA			P7
1	B12	2[47]0[48]BLK	ACGGCTACAAAAGGAGCCTTTAATGTGAGAAT			P7
1	C1	21[56]23[63]BLK	AGCTGATTGCCCTTCAGAGTCCACTATTAAAGGGTGCCGT			P7
1	C4	15[64]18[64]BLK	GTATAAGCCAACCCGTCGGATTCTGACGACAGTATCGGCCGCAAGGCG			
1	C5	13[64]15[63]BLK	TATATTTTGTCATTGCCTGAGAGTGGAAGATT			
1	C6	11[64]13[63]BLK	GATTTAGTCAATAAAGCCTCAGAGAACCCTCA		P3	
1	C7	9[64]11[63]BLK	CGGATTGCAGAGCTTAATTGCTGAAACGAGTA		P3	
1	C8	7[56]9[63]BLK	ATGCAGATACATAACGGGAATCGTCATAAATAAAGCAAAG			
1	C11	1[64]4[64]BLK	TTTATCAGGACAGCATCGGAACGACACCAACCTAAAACGAGGTCAATC			

1	C12	0[79]1[63]BLK	ACAACTTTCAACAGTTTCAGCGGATGTATCGG			P7
1	D1	23[64]22[80]BLK	AAAGCACTAAATCGGAACCCTAATCCAGTT			
1	D2	22[79]20[80]BLK	TGGAACAACCGCCTGGCCCTGAGGCCCGCT	P1		
1	D3	20[79]18[80]BLK	TTCCAGTCGTAATCATGGTCATAAAAGGGG	P1		
1	D4	18[79]16[80]BLK	GATGTGCTTCAGGAAGATCGCACAATGTGA			
1	D5	16[79]14[80]BLK	GCGAGTAAAAATATTTAAATTGTTACAAAG			
1	D6	14[79]12[80]BLK	GCTATCAGAAATGCAATGCCTGAATTAGCA			
1	D7	12[79]10[80]BLK	AAATTAAGTTGACCATTAGATACTTTTGCG			
1	D8	10[79]8[80]BLK	GATGGCTTATCAAAAAGATTAAGAGCGTCC			
1	D9	8[79]6[80]BLK	AATACTGCCCAAAAGGAATTACGTGGCTCA	P1		
1	D10	6[79]4[80]BLK	TTATACCACCAAATCAACGTAACGAACGAG	P1		
1	D11	4[79]2[80]BLK	GCGCAGACAAGAGGCAAAAGAATCCCTCAG			
1	D12	2[79]0[80]BLK	CAGCGAAACTTGCTTTCGAGGTGTTGCTAA			
1	E1	21[96]23[95]BLK	AGCAAGCGTAGGGTTGAGTGTTGTAGGGAGCC			
1	E2	19[96]21[95]BLK	CTGTGTGATTGCGTTGCGCTCACTAGAGTTGC	P1		
1	E3	17[96]19[95]BLK	GCTTTCCGATTACGCCAGCTGGCGGCTGTTTC	P1		
1	E4	15[96]17[95]BLK	ATATTTTGGCTTTCATCAACATTATCCAGCCA			
1	E5	13[96]15[95]BLK	TAGGTAAACTATTTTTGAGAGATCAAACGTTA			
1	E6	11[96]13[95]BLK	AATGGTCAACAGGCAAGGCAAAGAGTAATGTG			
1	E7	9[96]11[95]BLK	CGAAAGACTTTGATAAGAGGTCATATTTCGCA			
1	E8	7[96]9[95]BLK	TAAGAGCAAATGTTTAGACTGGATAGGAAGCC			
1	E9	5[96]7[95]BLK	TCATTCAGATGCGATTTTAAGAACAGGCATAG	P1		
1	E10	3[96]5[95]BLK	ACACTCATCCATGTTACTTAGCCGAAAGCTGC	P1		
1	E11	1[96]3[95]BLK	AAACAGCTTTTTGCGGGATCGTCAACACTAAA			
1	E12	0[111]1[95]BLK	TAAATGAATTTTCTGTATGGGATTAATTTCTT			
1	F1	23[96]22[112]BLK	CCCGATTTAGAGCTTGACGGGGAAAAAGAATA	P1		
1	F2	22[111]20[112]BLK	GCCCGAGAGTCCACGCTGGTTTGCAGCTAACT	P1		
1	F3	20[111]18[112]BLK	CACATTAAAATTGTTATCCGCTCATGCGGGCC			
1	F4	18[111]16[112]BLK	TCTTCGCTGCACCGCTTCTGGTGCGGCCTTCC			
1	F5	16[111]14[112]BLK	TGTAGCCATTAAAATTCGCATTAAATGCCGGA			
1	F6	14[111]12[112]BLK	GAGGGTAGGATTCAAAAGGGTGAGACATCCAA			
1	F7	12[111]10[112]BLK	TAAATCATATAACCTGTTTAGCTAACCTTTAA			
1	F8	10[111]8[112]BLK	TTGCTCCTTTCAAATATCGCGTTTGAGGGGGT	P1		
1	F9	8[111]6[112]BLK	AATAGTAAACACTATCATAACCCTCATTGTGA	P1		
1	F10	6[111]4[112]BLK	ATTACCTTTGAATAAGGCTTGCCCAAATCCGC			
1	F11	4[111]2[112]BLK	GACCTGCTCTTTGACCCCCAGCGAGGGAGTTA			
1	F12	2[111]0[112]BLK	AAGGCCGCTGATACCGATAGTTGCGACGTTAG			
1	G1	21[120]23[127]BLK	CCCAGCAGGCGAAAAATCCCTTATAAATCAAGCCGGCG			
1	G4	15[128]18[128]BLK	TAAATCAAAATAATTCGCGTCTCGGAAACCAGGCAAAGGGAAGG			
1	G5	13[128]15[127]BLK	GAGACAGCTAGCTGATAAATTAATTTTTGT			
1	G6	11[128]13[127]BLK	TTTGGGGATAGTAGTAGCATTAAAAGGCCG			
1	G7	9[128]11[127]BLK	GCTTCAATCAGGATTAGAGAGTTATTTTCA			
1	G8	7[120]9[127]BLK	CGTTTACCAGACGACAAAGAAGTTTTGCCATAATTCGA			
1	G11	1[128]4[128]BLK	TGACAACTCGCTGAGGCTTGCATTATACCAAGCGCGATGATAAA			
1	G12	0[143]1[127]BLK	TCTAAAGTTTTGTCGTCTTTCCAGCCGACAA			
1	H1	21[160]22[144]BLK	TCAATATCGAACCTCAAATATCAATTCCGAAA		P3	
1	H2	19[160]20[144]BLK	GCAATTCACATATTCCTGATTATCAAAGTGTA		P3	
1	H3	17[160]18[144]BLK	AGAAAACAAAGAAGATGATGAAACAGGCTGCG			
1	H4	15[160]16[144]BLK	ATCGCAAGTATGTAAATGCTGATGATAGGAAC			

1	H5	13[160]14[144]BLK	GTAATAAGTTAGGCAGAGGCATTTATGATATT			
1	H6	11[160]12[144]BLK	CCAATAGCTCATCGTAGGAATCATGGCATCAA		P3	
1	H7	9[160]10[144]BLK	AGAGAGAAAAAAATGAAAATAGCAAGCAAACT		P3	
1	H8	7[160]8[144]BLK	TTATTACGAAGAACTGGCATGATTGCGAGAGG			
1	H9	5[160]6[144]BLK	GCAAGGCCTCACCAGTAGCACCATGGGCTTGA			
1	H10	3[160]4[144]BLK	TTGACAGGCCACCACCAGAGCCGCGATTTGTA			
1	H11	1[160]2[144]BLK	TTAGGATTGGCTGAGACTCCTCAATAACCGAT		P3	
1	H12	0[175]0[144]BLK	TCCACAGACAGCCCTCATAGTTAGCGTAACGA		P3	
2	A1	23[128]23[159]BLK	AACGTGGCGAGAAAGGAAGGGAAACCAGTAA		P3	
2	A2	22[143]21[159]BLK	TCGGCAAATCCTGTTTGATGGTGGACCCTCAA		P3	
2	A3	20[143]19[159]BLK	AAGCCTGGTACGAGCCGGAAGCATAGATGATG			
2	A4	18[143]17[159]BLK	CAACTGTTGCGCCATTCGCCATTCAAACATCA			
2	A5	16[143]15[159]BLK	GCCATCAAGCTCATTTTTTAACCACAAATCCA			
2	A6	14[143]13[159]BLK	CAACCGTTTCAAATCACCATCAATTCGAGCCA		P3	
2	A7	12[143]11[159]BLK	TTCTACTACGCGAGCTGAAAAGGTTACCGCGC		P3	
2	A8	10[143]9[159]BLK	CCAACAGGAGCGAACCAGACCGGAGCCTTTAC			
2	A9	8[143]7[159]BLK	CTTTTGCAGATAAAAACCAAAATAAAGACTCC			
2	A10	6[143]5[159]BLK	GATGGTTTGAACGAGTAGTAAATTTACCATTA			
2	A11	4[143]3[159]BLK	TCATCGCCAACAAAGTACAACGGACGCCAGCA		P3	
2	A12	2[143]1[159]BLK	ATATTCGGAACCATCGCCCACGCAGAGAAGGA		P3	
2	B1	23[160]22[176]BLK	TAAAAGGGACATTCTGGCCAACAAAGCATC		P3	
2	B2	22[175]20[176]BLK	ACCTTGCTTGGTCAGTTGGCAAAGAGCGGA		P3	
2	B3	20[175]18[176]BLK	ATTATCATTCAATATAATCCTGACAATTAC			
2	B4	18[175]16[176]BLK	CTGAGCAAAAATTAATTACATTTTGGGTTA			
2	B5	16[175]14[176]BLK	TATAACTAACAAAGAACGCGAGAACGCCAA			
2	B6	14[175]12[176]BLK	CATGTAATAGAATATAAAGTACCAAGCCGT		P3	
2	B7	12[175]10[176]BLK	TTTTATTTAAGCAAATCAGATATTTTTTGT		P3	
2	B8	10[175]8[176]BLK	TTAACGTCTAACATAAAAACAGGTAACGGA			
2	B9	8[175]6[176]BLK	ATACCCAACAGTATGTTAGCAAATTAGAGC			
2	B10	6[175]4[176]BLK	CAGCAAAAGGAAACGTCACCAATGAGCCGC			
2	B11	4[175]2[176]BLK	CACCAGAAAGGTTGAGGCAGGTCATGAAAG		P3	
2	B12	2[175]0[176]BLK	TATTAAGAAGCGGGGTTTTGCTCGTAGCAT		P3	
2	C1	21[184]23[191]BLK	TCAACAGTTGAAAGGAGCAAATGAAAAATCTAGAGATAGA			
2	C4	15[192]18[192]BLK	TCAAATATAACCTCCGGCTTAGGTAACAATTTCATTTGAAGGCGAATT			
2	C5	13[192]15[191]BLK	GTAAAGTAATCGCCATATTTAACAAAACTTTT			
2	C6	11[192]13[191]BLK	TATCCGGTCTCATCGAGAACAAGCGACAAAAG			
2	C7	9[192]11[191]BLK	TTAGACGGCCAAATAAGAAACGATAGAAGGCT			
2	C8	7[184]9[191]BLK	CGTAGAAAATACATACCGAGGAAACGCAATAAGAAGCGCA			
2	C11	1[192]4[192]BLK	GCGGATAACCTATTATTCTGAAACAGACGATTGGCCTTGAAGAGCCAC			
2	C12	0[207]1[191]BLK	TCACCAGTACAAACTACAACGCCTAGTACCAG			
2	D1	23[192]22[208]BLK	ACCCTTCTGACCTGAAAGCGTAAGACGCTGAG			
2	D2	22[207]20[208]BLK	AGCCAGCAATTGAGGAAGGTTATCATCATTTT	P1		
2	D3	20[207]18[208]BLK	GCGGAACATCTGAATAATGGAAGGTACAAAAT	P1		
2	D4	18[207]16[208]BLK	CGCGCAGATTACCTTTTTTAATGGGAGAGACT			
2	D5	16[207]14[208]BLK	ACCTTTTTATTTTAGTTAATTTCATAGGGCTT			
2	D6	14[207]12[208]BLK	AATTGAGAATTCTGTCCAGACGACTAAACCAA			
2	D7	12[207]10[208]BLK	GTACCGCAATTCTAAGAACGCGAGTATTATTT			
2	D8	10[207]8[208]BLK	ATCCCAATGAGAATTAACTGAACAGTTACCAG			
2	D9	8[207]6[208]BLK	AAGGAAACATAAAGGTGGCAACATTATCACCG	P1		

2	D10	6[207]4[208]BLK	TCACCGACGCACCGTAATCAGTAGCAGAACCG	P1		
2	D11	4[207]2[208]BLK	CCACCCTCTATTCACAAACAAATACCTGCCTA			
2	D12	2[207]0[208]BLK	TTTCGGAAGTGCCGTCGAGAGGGTGAGTTTCG			
2	E1	21[224]23[223]BLK	CTTTAGGGCCTGCAACAGTGCCAATACGTG			
2	E2	19[224]21[223]BLK	CTACCATAGTTTGAGTAACATTTAAAATAT	P1		
2	E3	17[224]19[223]BLK	CATAAATCTTTGAATACCAAGTGTTAGAAC	P1		
2	E4	15[224]17[223]BLK	CCTAAATCAAAATCATAGGTCTAAACAGTA			
2	E5	13[224]15[223]BLK	ACAACATGCCAACGCTCAACAGTCTTCTGA			
2	E6	11[224]13[223]BLK	GCGAACCTCCAAGAACGGGTATGACAATAA			
2	E7	9[224]11[223]BLK	AAAGTCACAAAATAAACAGCCAGCGTTTTA			
2	E8	7[224]9[223]BLK	AACGCAAAGATAGCCGAACAAACCCTGAAC			
2	E9	5[224]7[223]BLK	TCAAGTTTCATTAAAGGTGAATATAAAAGA	P1		
2	E10	3[224]5[223]BLK	TTAAAGCCAGAGCCGCCACCCTCGACAGAA	P1		
2	E11	1[224]3[223]BLK	GTATAGCAAACAGTTAATGCCCAATCCTCA			
2	E12	0[239]1[223]BLK	AGGAACCCATGTACCGTAACACTTGATATAA			
2	F1	23[224]22[240]BLK	GCACAGACAATATTTTTGAATGGGGTCAGTA	P1		
2	F2	22[239]20[240]BLK	TTAACACCAGCACTAACAACTAATCGTTATTA	P1		
2	F3	20[239]18[240]BLK	ATTTTAAAATCAAAATTATTTGCACGGATTCG			
2	F4	18[239]16[240]BLK	CCTGATTGCAATATATGTGAGTGATCAATAGT			
2	F5	16[239]14[240]BLK	GAATTTATTTAATGGTTTGAAATATTCTTACC			
2	F6	14[239]12[240]BLK	AGTATAAAGTTCAGCTAATGCAGATGTCTTTC			
2	F7	12[239]10[240]BLK	CTTATCATTCCCGACTTGCGGGAGCCTAATTT			
2	F8	10[239]8[240]BLK	GCCAGTTAGAGGGTAATTGAGCGCTTTAAGAA	P1		
2	F9	8[239]6[240]BLK	AAGTAAGCAGACACCACGGAATAATATTGACG	P1		
2	F10	6[239]4[240]BLK	GAAATTATTGCCTTTAGCGTCAGACCGGAACC			
2	F11	4[239]2[240]BLK	GCCTCCCTCAGAATGGAAAGCGCAGTAACAGT			P7
2	F12	2[239]0[240]BLK	GCCCGTATCCGGAATAGGTGTATCAGCCCAAT			P7
2	G1	21[248]23[255]BLK	AGATTAGAGCCGTCAAAAAACAGAGGTGAGGCCTATTAGT			
2	G4	15[256]18[256]BLK	GTGATAAAAAGACGCTGAGAAGAGATAACCTTGCTTCTGTTCGGGAGA			
2	G5	13[256]15[255]BLK	GTTTATCAATATGCGTTATACAAACCGACCGT			
2	G6	11[256]13[255]BLK	GCCTTAAACCAATCAATAATCGGCACGCGCCT			
2	G7	9[256]11[255]BLK	GAGAGATAGAGCGTCTTTCCAGAGGTTTTGAA			
2	G8	7[248]9[255]BLK	GTTTATTTTGTCACAATCTTACCGAAGCCCTTTAATATCA			
2	G11	1[256]4[256]BLK	CAGGAGGTGGGGTCAGTGCCTTGAGTCTCTGAATTTACCGGGAACCAG			
2	G12	0[271]1[255]BLK	CCACCCTCATTTTCAGGGATAGCAACCGTACT			P7
2	H1	23[256]22[272]BLK	CTTTAATGCGCGAACTGATAGCCCCACCAG			
2	H2	22[271]20[272]BLK	CAGAAGATTAGATAATACATTTGTCGACAA			
2	H3	20[271]18[272]BLK	CTCGTATTAGAAATTGCGTAGATACAGTAC			
2	H4	18[271]16[272]BLK	CTTTTACAAAATCGTCGCTATTAGCGATAG			
2	H5	16[271]14[272]BLK	CTTAGATTTAAGGCGTTAAATAAAGCCTGT			
2	H6	14[271]12[272]BLK	TTAGTATCACAATAGATAAGTCCACGAGCA			
2	H7	12[271]10[272]BLK	TGTAGAAATCAAGATTAGTTGCTCTTACCA			
2	H8	10[271]8[272]BLK	ACGCTAACACCCACAAGAATTGAAAATAGC			
2	H9	8[271]6[272]BLK	AATAGCTATCAATAGAAAATTCAACATTCA			
2	H10	6[271]4[272]BLK	ACCGATTGTCGGCATTTTCGGTCATAATCA			P7
2	H11	4[271]2[272]BLK	AAATCACCTTCCAGTAAGCGTCAGTAATAA			P7
2	H12	2[271]0[272]BLK	GTTTTAACTTAGTACCGCCACCCAGAGCCA			P7

Supplementary Tab. 3 | Biotinylated staple strands

Position	Name	Sequence	Modification
C02	18[63]20[56]BIOTIN	ATTAAGTTTACCGAGCTCGAATTCGGGAAACCTGTCGTGC	5' - Biotin
C09	4[63]6[56]BIOTIN	ATAAGGGAACCGGATATTCATTACGTCAGGACGTTGGGAA	5' - Biotin
G02	18[127]20[120]BIOTIN	GCGATCGGCAATTCCACACAACAGGTGCCTAATGAGTG	5' - Biotin
G09	4[127]6[120]BIOTIN	TTGTGTCGTGACGAGAAACACCAAATTTCAACTTTAAT	5' - Biotin
K02	18[191]20[184]BIOTIN	ATTCATTTTTGTTTGGATTATACTAAGAAACCACCAGAAG	5' - Biotin
K09	4[191]6[184]BIOTIN	CACCCTCAGAAACCATCGATAGCATTGAGCCATTTGGGAA	5' - Biotin
O02	18[255]20[248]BIOTIN	AACAATAACGTAAAACAGAAATAAAAATCCTTTGCCCGAA	5' - Biotin
O09	4[255]6[248]BIOTIN	AGCCACCACTGTAGCGCGTTTTCAAGGGAGGGAAGGTAAA	5' - Biotin

Supplementary Tab. 4 | DNA-PAINT docking site sequences

Name	Sequence	Modification
P1 docking strand	TTATACATCTA	-
P3 docking strand	TTTCTTCATTA	-
P7 docking strand	TTAATTGAGTA	-

Supplementary Tab. 5 | DNA-PAINT imager sequences

Name	Sequence	Modification
Imager P1 – ATTO 643	CTAGATGTAT	3' – ATTO 643
Imager P3 – Cy3B	GTAATGAAGA	3' – Cy3B
Imager P7 – Alexa Fluor 680	GTACTCAATT	3' – Alexa Fluor 680
Imager P7 – ATTO 680	GTACTCAATT	3' – ATTO 680

Supplementary Tab. 6 | Experimental conditions for sample Letter O

Microscope setting	Condition
Frames	5000
Exposure time	200 ms
Excitation laser	642 nm
Laser Power	130 mW

Sample	Condition
Imager sequence	P1
Imager concentration	3 nM
Imaging buffer	B with PCA/PCD/TX
Dye	ATTO 643

Supplementary Tab. 7 | Experimental conditions for sample Letter T

Microscope setting	Condition
Frames	5000
Exposure time	200 ms
Excitation laser	560 nm
Laser Power	100 mW

Sample	Condition
Imager sequence	P3
Imager concentration	3 nM
Imaging buffer	B with PCA/PCD/TX
Dye	Cy3B

Supplementary Tab. 8 | Experimental conditions for sample Letter L

Microscope setting	Condition
Frames	7500
Exposure time	200 ms
Excitation laser	642 nm
Laser Power	250 mW

Sample	Condition
Imager sequence	P7
Imager concentration	4 nM
Imaging buffer	B
Dye	ATT= 680

Supplementary Tab. 9 | Experimental conditions for combined sample Letter O, Letter T, Letter L

Microscope setting	Condition
Frames	30 000
Exposure time	250 ms
Excitation laser	560nm, 642 nm
Laser Power	100 mW, 200 mW

Sample	Condition
Imager sequence	P1, P3, P7
Imager concentration	1 nM, 1 nM, 4 nM
Imaging buffer	B with PCA/PCD/TX
Dye	ATTO 643, Cy3B, Alexa Fluor 680
